# The Coordinated Action of Calcineurin and Cathepsin D Protects Against α-Synuclein Toxicity

**DOI:** 10.3389/fnmol.2017.00207

**Published:** 2017-06-30

**Authors:** Andreas Aufschnaiter, Lukas Habernig, Verena Kohler, Jutta Diessl, Didac Carmona-Gutierrez, Tobias Eisenberg, Walter Keller, Sabrina Büttner

**Affiliations:** ^1^Institute of Molecular Biosciences, University of GrazGraz, Austria; ^2^Department of Molecular Biosciences, The Wenner-Gren Institute, Stockholm UniversityStockholm, Sweden

**Keywords:** α-synuclein, Parkinson’s disease, cathepsin D, Pep4, calcineurin, cytosolic acidification, pH homeostasis, vacuole

## Abstract

The degeneration of dopaminergic neurons during Parkinson’s disease (PD) is intimately linked to malfunction of α-synuclein (αSyn), the main component of the proteinaceous intracellular inclusions characteristic for this pathology. The cytotoxicity of αSyn has been attributed to disturbances in several biological processes conserved from yeast to humans, including Ca^2+^ homeostasis, general lysosomal function and autophagy. However, the precise sequence of events that eventually results in cell death remains unclear. Here, we establish a connection between the major lysosomal protease cathepsin D (CatD) and the Ca^2+^/calmodulin-dependent phosphatase calcineurin. In a yeast model for PD, high levels of human αSyn triggered cytosolic acidification and reduced vacuolar hydrolytic capacity, finally leading to cell death. This could be counteracted by overexpression of yeast CatD (Pep4), which re-installed pH homeostasis and vacuolar proteolytic function, decreased αSyn oligomers and aggregates, and provided cytoprotection. Interestingly, these beneficial effects of Pep4 were independent of autophagy. Instead, they required functional calcineurin signaling, since deletion of calcineurin strongly reduced both the proteolytic activity of endogenous Pep4 and the cytoprotective capacity of overexpressed Pep4. Calcineurin contributed to proper endosomal targeting of Pep4 to the vacuole and the recycling of the Pep4 sorting receptor Pep1 from prevacuolar compartments back to the trans-Golgi network. Altogether, we demonstrate that stimulation of this novel calcineurin-Pep4 axis reduces αSyn cytotoxicity.

## Introduction

Parkinson’s disease (PD) is an age-associated neurodegenerative disorder characterized by the progressive loss of dopaminergic neurons in the *substantia nigra pars compacta*. Malfunction of α-synuclein (αSyn) is thought to play a key role in PD pathophysiology, since this protein constitutes the main component of the proteinaceous deposits, so-called Lewy bodies, which present a hallmark of PD (Spillantini et al., 1997; Baba et al., 1998). Furthermore, mutations as well as multiplications of the gene coding for αSyn are linked to familial PD (Polymeropoulos et al., 1997; Singleton et al., 2003).

Although the physiological function of αSyn is not fully understood yet, it abundantly appears in presynaptic terminals and is supposed to promote SNARE-complex assembly (Burré et al., 2010). High levels of αSyn or pathological point mutants result in an impairment of vesicle trafficking (especially ER-to-Golgi complex trafficking; Cooper et al., 2006; Thayanidhi et al., 2010), mitochondrial defects and oxidative stress (Büttner et al., 2008; Bose and Beal, 2016), deficiency in proteasomal degradation (Chung et al., 2001), imbalance of Ca^2+^ homeostasis (Büttner et al., 2013a; Rcom-H’cheo-Gauthier et al., 2016), lysosomal dysfunction (Tofaris, 2012; Bourdenx et al., 2014), and insufficient autophagy (Winslow et al., 2010; Lynch-Day et al., 2012). Vice versa, induction of autophagy by different means reduces the toxic effects of αSyn in diverse model systems of PD (Decressac et al., 2013; Hebron et al., 2013; Chen et al., 2014). Interestingly, various proteins governing Ca^2+^ homeostasis have been shown to influence autophagic processes. However, this interplay seems rather complex, and controversial studies describe either an inhibition or an activation of autophagy by Ca^2+^-regulating proteins or by changes in cytosolic and lumenal Ca^2+^ levels (Høyer-Hansen et al., 2007; Vicencio et al., 2009; Gastaldello et al., 2010; Grotemeier et al., 2010; Harr et al., 2010). In this line, both excess and insufficient activity of the Ca^2+^/calmodulin-dependent phosphatase calcineurin have been shown to cause neurotoxicity (Foster et al., 2001; Zeng et al., 2001; Wu et al., 2004; Sklar, 2006; Bahi et al., 2009), while an intermediate activation of this enzyme reduced αSyn cytotoxicity in a yeast model of PD (Caraveo et al., 2014). Combined, these results point towards a complex connection between Ca^2+^ signaling and autophagy in PD.

Considering the formation of αSyn aggregates in the pathogenesis of PD and the protective role of autophagy in αSyn cytotoxicity, enhancing the lysosomal degradation of αSyn oligomers might represent an attractive approach for therapeutic interventions in PD. Indeed, overexpression of the lysosomal aspartyl protease cathepsin D (CatD) was able to alleviate αSyn-induced cellular demise (Qiao et al., 2008; Sevlever et al., 2008). In line, high levels of αSyn reduced the proteolytic activity of endogenous CatD (Matrone et al., 2016). However, even though CatD seems to act as the major protease involved in the degradation of αSyn and high CatD levels mostly provided neuroprotection (Qiao et al., 2008; Sevlever et al., 2008; Matrone et al., 2016), pharmacological inhibition of this lysosomal aspartyl protease has also been demonstrated to decrease αSyn aggregation propensity and toxicity (Takahashi et al., 2007).

While both calcineurin and CatD have been connected to neurotoxic events in general and to αSyn-associated cellular demise in particular, an interplay between these two proteins has never been shown. Using a well-established yeast model of PD (Cooper et al., 2006; Büttner et al., 2008, 2013a,b; Tardiff et al., 2013; Menezes et al., 2015), we studied a potential interaction of calcineurin and Pep4, the yeast ortholog of CatD, in αSyn-mediated cytotoxicity. We connect calcineurin signaling to Pep4 activity, the trafficking and recycling of its vacuolar sorting receptor Pep1 and, moreover, to the cytoprotective effects of high levels of Pep4. Heterologous expression of human αSyn resulted in impaired pH homeostasis, mislocalization of Pep1 and Pep4 and a reduction of Pep4 activity. Overexpression of Pep4 diminished αSyn monomers, oligomers and aggregates in an autophagy-independent way and inhibited αSyn-mediated cytosolic acidification and cell death. These cytoprotective effects of Pep4 required functional calcineurin signaling.

## Materials and Methods

### *Saccharomyces Cerevisiae* Strains and Genetics

Experiments were carried out in BY4741 (*MAT***a**; *his3*Δ1; *leu2*Δ0; *met15*Δ0; *ura3*Δ0) obtained from Euroscarf. Heterologous expression of C-terminally FLAG-tagged human αSyn under the control of a GAL10 promoter was performed using a pESC-URA plasmid (Stratagene). Therefore, a previously published αSyn-construct in pESC-HIS (Büttner et al., 2008, 2013b) was cut with *Spe*I and *Cla*I and ligated into pESC-URA. All oligonucleotides used in this study are listed in Supplementary Table S1. Constructs for overexpression of Vma1 and Vph2 in pESC-HIS were kindly provided by Ruckenstuhl et al. (2014). For overexpression of FLAG-tagged wild type Pep4 (Pep4^WT^) and the double point mutant (D109A, D294A) of Pep4 (Pep4^DPM^) under a galactose promoter, previously published constructs in pESC-HIS were used (Carmona-Gutierrez et al., 2011). The *PMA1* gene was amplified and cloned with *Not*I and *Spe*I into pESC-HIS and *PEP1* was inserted into a pESC-LEU plasmid using *Not*I. To visualize αSyn localization and aggregation, the C-terminally tagged variant αSyn-GFP was amplified from pUG23 (Zabrocki et al., 2005) and cloned with *Spe*I and *Cla*I into pESC-URA.

Transformation of plasmids into yeast cells was performed using the standard lithium acetate method (Gietz and Woods, 2002). Deletion mutants were either obtained from Euroscarf or generated via homologous recombination according to (Janke et al., 2004; for a detailed list see Supplementary Table S2). All oligonucleotides and plasmids used for this approach are listed in Supplementary Table S1. At least four different clones were tested after plasmid transformation or genomic replacement to rule out clonogenic variations.

### Media and Culturing Conditions

Yeast strains were grown in synthetic complete (SC) medium containing 0.17% yeast nitrogen base (Difco, BD Biosciences), 0.5% (NH_4_)_2_SO_4_ and 30 mg/L of all amino acids (except 80 mg/L histidine and 200 mg/L leucine), 30 mg/L adenine and 320 mg/L uracil with 2% D-glucose (SCD) or 2% D-galactose (SCG) for GAL10-driven expression of α-Syn, Pep4^WT^ or Pep4^DPM^. All media were prepared with double distilled water and subsequently autoclaved (25 min, 121°C, 210 kPa). Amino acid mixtures were sterilized separately as 10× stocks and added after autoclaving. For solid media, 2% agar was admixed. Full media (yeast extract peptone dextrose, YEPD) agar plates contained 1% yeast extract (Bacto, BD Biosciences), 2% peptone (Bacto, BD Biosciences) and 4% D-glucose.

Experiments were performed using overnight cultures grown in SCD for 16–20 h at 28°C and 145 rpm. These cultures were inoculated in 10 mL SCD (in 100 mL Erlenmeyer flasks) to an OD_600_ of 0.1 and grown to OD_600_ 0.3. Subsequently, cells were transferred into 96-well deep well plates (VWR), whereat 500 μL of cell culture was used per well. After pelleting, cells were shifted to 500 μL SCG per well for induction of expression. For inhibition of Pep4 enzymatic activity, 50 μM pepstatin A dissolved in DMSO (Sigma) were added to cells upon galactose-mediated induction of expression.

### Analysis of Cell Death

To measure loss of membrane integrity, approximately 2 × 10^6^ cells were collected in 96-well plates via centrifugation at indicated time points after induction of expression and resuspended in 250 μL phosphate buffered saline (PBS, 25 mM potassium phosphate; 0.9% NaCl; adjusted to pH 7.2) containing 100 μg/L propidium iodide (PI). Cells were incubated for 10 min in the dark, pelleted via centrifugation and washed once in 250 μL PBS. PI staining was quantified via flow cytometry (BD LSR Fortessa), analyzing 30,000 cells with BD FACSDivia software.

### Immunoblotting and *In Vivo* Crosslinking

Whole cell extracts were generated by chemical lysis. Cells equivalent to an OD_600_ of three (for general immunoblotting) or an OD_600_ of eight (for detection of αSyn oligomers) were harvested 24 h after induction of expression, resuspended in 200 μL of 0.1 M NaOH and incubated shaking with 1400 rpm and 21°C for 5 min. After centrifugation with 4000 rpm for 5 min, pellets were resuspended in 150 μL 1× Laemmli buffer (50 mM Tris-HCl; 2% SDS; 10% glycerol; 0.1% bromophenol blue; 100 mM 2-mercaptoethanol; adjusted to pH 6.8) and again shaken with 1400 rpm and 21°C for 5 min. Of note, samples for detection of αSyn oligomers were prepared with 1× Laemmli buffer without 2-mercaptoethanol (semi-native approach). Samples were centrifuged with 13,000 rpm for 1 min and 15 μL of the supernatant was used for standard SDS-PAGE. To detect αSyn oligomers, polyacrylamide gels without SDS were applied and electrophoresis was performed at 4°C (semi-native approach). Immunoblotting was performed using standard protocols with antibodies directed against αSyn (Sigma, S3062), FLAG epitope (Sigma; F3165), influenza hemagglutinin protein (HA epitope; Sigma H3663), Pep1 (Abcam; ab113690), yeast glyceraldehyde 3-phosphate dehydrogenase (GAPDH; gift from Sepp Kohlwein, University of Graz) and the respective peroxidase-conjugated affinity-purified secondary antibodies (Sigma). A ChemiDoc™ Touch Imaging System (Bio-Rad) was used for detection, and subsequent densitometric quantification was performed with Image Lab 5.2 Software (Bio-Rad).

*In vivo* crosslinking experiments were performed with adapted protocols according to Klockenbusch and Kast (2010). All washing steps were accomplished with and all reagents were solubilized in 0.1 M sodium phosphate buffer (0.02 M Na_2_HPO_4_; 0.08 M NaH_2_PO_4_; adjusted to pH 7.4). In brief, cells equivalent to an OD_600_ of five were harvested 24 h after induction of expression, washed once and resuspended in 1 mL of 1% formaldehyde. Of note, a negative control for every sample was resuspended in 1 mL buffer. Cells were incubated for 9 min and centrifuged for 1 min with 13,000 rpm. 1 mL of 1.25 M glycine was added to stop the reaction and incubated for 5 min. Cells were washed five times, followed by lysis and immunoblotting, conducted as described for semi-native detection of αSyn oligomers.

For densitometric quantification, signals were normalized to the respective GAPDH signal and fold change of αSyn oligomers upon expression of Pep4^WT^ or Pep4^DPM^ were plotted.

Indicated molecular weights in all shown immunoblots represent the apparent molecular weight (kDa) determined with a PageRuler prestained protein ladder (ThermoFisher Scientific) as indicated by the manufacturers migration pattern.

### Pep4 Activity Assay

To measure the enzymatic activity of Pep4, a fluorometric CatD activity assay kit from Abcam (ab65302) was used and the protocol was adapted for yeast samples. Briefly, 2 × 10^6^ cells were harvested at specified time points after induction of expression. Protein extraction was performed with glass beads and the supplied CD cell lysis buffer and the resulting protein concentration was determined via a Bradford assay (Bio-Rad). Afterwards, 0.1 μg protein was used for the CatD activity assay. Reactions were incubated for 2 h at 28°C and the fluorescence signal was measured with a Tecan Genios pro microplate reader (ex: 328 nm, em: 460 nm) and plotted as fold change compared to the empty vector control. Of note, a Δ*pep4* strain was used as background control in each assay, being subtracted from presented Pep4 activity measurements.

### Assessment of Cytosolic Calcium Levels with Aequorin

Measurement of basal cytosolic calcium levels [Ca^2+^]_cyt_ was performed as described in Büttner et al. (2013a). Briefly, galactose-driven expression of αSyn was established in yeast strains carrying the vector pEVP11/AEQ89, coding for the bioluminescent reporter protein aequorin (kind gift from Kyle W. Cunningham) and cells were cultivated as described above. At indicated time points, approximately 1 × 10^8^ cells were transferred into a 96-well plate and harvested by centrifugation. The cell pellet was resuspended in 200 μL SCD medium (plus 4 μM colenterazine h, ThermoFisher Scientific) and incubated for 1 h in the dark. Excess colenterazine h was removed by washing once in SCD followed by a further incubation step of 30 min. The basal luminescence signal was measured in 0.5 s intervals for 20 s with an Orion II Microplate Luminometer (Berthold Detection Systems). The obtained signal was normalized to OD_600_ of each well and aequorin protein levels in each transformant.

### Quinacrine Staining to Visualize Acidic Cell Compartments

Approximately 1 × 10^8^ cells were harvested at indicated time points after induction of expression and washed with 500 μL of YEPD containing 100 mM HEPES (pH 7.6). After centrifugation, the pellet was resuspended in 500 μL of YEPD with 100 mM HEPES (pH 7.6) and 400 μM quinacrine. After incubation for 10 min at 28°C and 145 rpm, samples were incubated 5 min on ice. After centrifugation, cells were washed twice in 500 μL ice-cold HEPES buffer supplemented with 2% D-glucose. For PI co-staining, 500 μL of HEPES buffer with 2% D-glucose and 100 μg/L PI was added and incubated 10 min in the dark on ice. Cells were analyzed using a Zeiss Axioskop epifluorescence microscope. For quantification, 300–700 cells per genotype and experiment were manually counted.

### MDY-64 Staining for Visualizing Vacuolar Morphology

Approximately 1 × 10^6^ cells from cultures were harvested at indicated time points after induction of expression and washed in 500 μL 10 mM HEPES buffer (pH 7.4) containing 5% D-glucose. Cells were resuspended in the same buffer containing 10 μM MDY-64 (Molecular Probes) and incubated for 3 min at room temperature in the dark. Subsequently, cells were washed in HEPES buffer and analyzed with a Zeiss Axioskop epifluorescence microscope. For quantification, 350–650 cells per genotype and experiment were manually counted.

### CMAC Staining of Vacuoles

Approximately 1 × 10^6^ cells from cultures were harvested at indicated time points after induction of expression and resuspended in 10 mM HEPES buffer (pH 7.4; supplemented with 5% glucose). Afterwards, CellTracker Blue CMAC (Molecular Probes) was added to a final concentration of 100 μM, cells were incubated in the dark for 20 min and subsequently analyzed with a Zeiss Axioskop epifluorescence microscope. For PI co-staining, PI (final concentration of 100 μg/L) was added to the CMAC staining solution. For analysis of vacuolar membrane permeabilization 2.5 μg/mL tunicamycin (Sigma) dissolved in DMSO was added to cells after the shift to galactose as positive control (adapted from Kim et al., 2012).

### Quantitative Real-Time PCR

To determine mRNA levels, total RNA was extracted from respective strains (OD_600_ 1) after 24 h of αSyn expression using TRIzol reagent (Thermo Scientific). Briefly, the cell pellet was resuspended in 500 μL TRIzol plus approximately 100 μl glass beads (500 μm diameter) and the samples were lysed mechanically in three cycles of 1 min. The subsequent steps were employed as described in the manual. Integrity of the isolated RNA was validated by visualizing rRNAs on an agarose gel (adapted from Aranda et al., 2012). Contaminating DNA was removed with the DNA-free Kit (Ambion) and the RNA concentration was determined spectrometrically with a NanoDrop (ND 1000 Spectrophotometer). 3.5 μg of total RNA were reverse transcribed using the M-MLV Reverse Transcriptase RNase H- (Solis Biodyne) and random hexamer primers (Thermo Scientific). Obtained cDNA was diluted accordingly and the actual q-RT-PCR was performed with the 5× HOT FIREPol EvaGreen qPCR Supermix (Solis Biodyne). Primers used in these experiments are listed in Supplementary Table S1. First, standard curve experiments were performed to ensure primer efficiency lying between 90% and 110%. The cDNA was used in duplicates for quantitative PCR amplification and the relative gene expression levels were calculated with the Comparative C_T_ Method (Livak and Schmittgen, 2001) with *UBC6* used as housekeeping gene for normalization.

### Statistical Analysis

To disprove the null-hypothesis (no difference between conditions), significance and *p*-values were calculated using one-way ANOVA, corrected with a Bonferroni *post hoc* test for one variable (type of expression). Significance is indicated with asterisks: ***p < 0.001, **p < 0.01, *p < 0.05. Statistics were conducted with Origin Pro 2016 (OriginLab) and figures were prepared with Origin Pro 2016 and Adobe Illustrator CS6 (Adobe). Microscopic pictures were processed with Fiji (Schindelin et al., 2012).

## Results

### High Levels of Pep4 Protect Against αSyn Cytotoxicity

**Figure 1:**
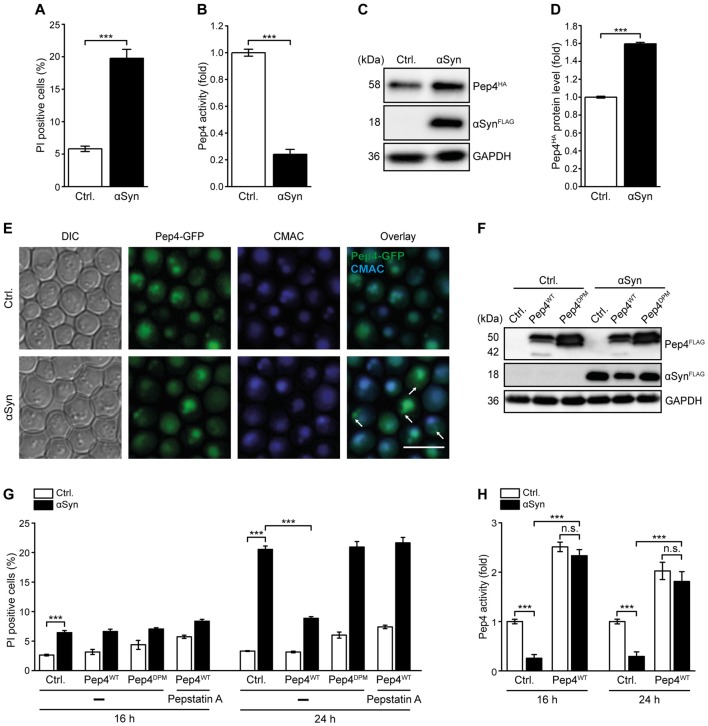
Pep4 overexpression counteracts αSyn-mediated defects of vacuolar proteolytic capacity and cell death. **(A)** Flow cytometric quantification of loss of membrane integrity, indicated by propidium iodide (PI) staining of cells expressing human α-synuclein (αSyn) for 24 h or harboring the corresponding vector control (Ctrl.). Means ± SEM; *n* = 28. **(B)** Measurement of Pep4 proteolytic activity in protein extracts 16 h after induction of αSyn expression in cells described above. Values obtained for Δ*pep4* cells have been subtracted as background, followed by normalization to the average of signals from Ctrl. cells. Means ± SEM; *n* = 16. **(C,D)** Immunoblot analysis of protein extracts from cells harboring chromosomally HA-tagged Pep4 and expressing αSyn for 16 h or harboring the corresponding vector control. A representative immunoblot **(C)** and quantification of Pep4-HA levels **(D)** are shown. Blots were probed with antibodies against HA epitope and glyceraldehyde 3-phosphate dehydrogenase (GAPDH) as a loading control. Means ± SEM; *n* = 7. **(E)** Representative micrographs of cells with chromosomally GFP-tagged Pep4 expressing αSyn for 24 h or harboring the corresponding vector control. CMAC counterstaining was performed to visualize vacuoles. White arrows indicate localization of Pep4 in prevacuolar compartments. **(F)** Immunoblot analysis of protein extracts from cells co-expressing αSyn and wild type Pep4 (Pep4^WT^) or the inactive double point mutant of Pep4 (Pep4^DPM^) for 24 h or harboring the corresponding vector controls. Blots were probed with antibodies directed against FLAG epitope to detect FLAG-tagged αSyn, Pep4^WT^ and Pep4^DPM^ and against GAPDH as loading control. **(G)** Flow cytometric quantification of loss of membrane integrity via PI staining of cells described in **(F)** at indicated time points. Cells were either treated with 50 μM pepstatin A or with DMSO (−). Means ± SEM; *n* ≥ 4. **(H)** Measurement of Pep4 proteolytic activity in protein extracts of cells expressing αSyn with and without co-expression of Pep4^WT^ for 16 h and 24 h, respectively, or harboring the corresponding vector controls. Values obtained for Δ*pep4* cells have been subtracted as background. Means ± SEM; *n* = 8. n.s. not significant, ***p < 0.001; Scale bar represents 5 μm.

General lysosomal dysfunction as well as alterations in CatD levels and activity are implicated in the pathogenesis of PD (Qiao et al., 2008; Sevlever et al., 2008; Manzoni and Lewis, 2013; Matrone et al., 2016; Moors et al., 2016). To further analyze the impact of αSyn on lysosomal integrity in general and on CatD function in particular, we used a yeast model for PD based on the heterologous expression of human αSyn. While αSyn did not affect cell proliferation, it caused an increase of cell death in early stationary phase, as indicated by PI staining (Figure 1A). Biochemical quantification of yeast CatD (Pep4) proteolytic capacity in these cells revealed a massive reduction of Pep4 enzymatic activity upon αSyn expression (Figure 1B). Values obtained for Δ*pep4* cells have been subtracted as background. Performing immunoblotting, we observed an increase in Pep4 protein levels upon αSyn expression (Figures 1C,D), suggesting a compensatory upregulation of Pep4 as a futile cellular attempt to counterbalance the αSyn-triggered loss of Pep4 proteolytic activity. Pep4/CatD has been shown to be released into the cytosol as a result of vacuolar/lysosomal membrane permeabilization during acetic acid-induced apoptosis in yeast and mammalian tumor cell lines (Pereira et al., 2010; Marques et al., 2013). Thus, we analyzed the effects of αSyn on the integrity of the limiting vacuolar membrane and the localization of Pep4. However, αSyn did neither induce vacuolar membrane permeabilization (Supplementary Figure S1) nor a detectable vacuolar release of a Pep4-GFP chimera into the cytosol (Figure 1E). Instead, the larger fraction of Pep4-GFP was still targeted to the vacuole, visualized via CellTracker Blue CMAC, a dye that is rapidly sequestered into the vacuolar lumen. Interestingly, expression of αSyn led to the accumulation of Pep4 in prevacuolar compartments in some cells, suggesting that αSyn interferes with the trafficking of this protease from the trans-Golgi network to the vacuole, which might in part account for the observed reduction of Pep4 activity. Thus, we overexpressed Pep4 to antagonize the αSyn-induced decrease of vacuolar catabolic activity. Indeed, co-expression of Pep4^WT^ was sufficient to prevent cell death caused by αSyn upon entry into stationary phase (Figures 1F,G). This cytoprotection required Pep4 protease activity, as the proteolytically inactive double point mutant Pep4^DPM^ (Carmona-Gutierrez et al., 2011) showed no effect (Figures 1F,G). In addition, treatment with the CatD inhibitor pepstatin A completely eliminated cytoprotection (Figure 1G). Measurement of Pep4 activity revealed that high levels of Pep4^WT^ not only led to a general increase of its enzymatic activity but also abrogated the αSyn-mediated reduction of proteolytic capacity (Figure 1H). Interestingly, only a minor fraction of cells died after 16 h of αSyn expression, even though Pep4 activity was already reduced to only 0.2-fold of WT levels. This indicates that the decrease of Pep4 activity during exponential growth preceded the actual onset of cell death in early stationary phase (Figures 1G,H).

### αSyn Triggers Cytosolic Acidification and Alters Vacuolar Morphology

**Figure 2:**
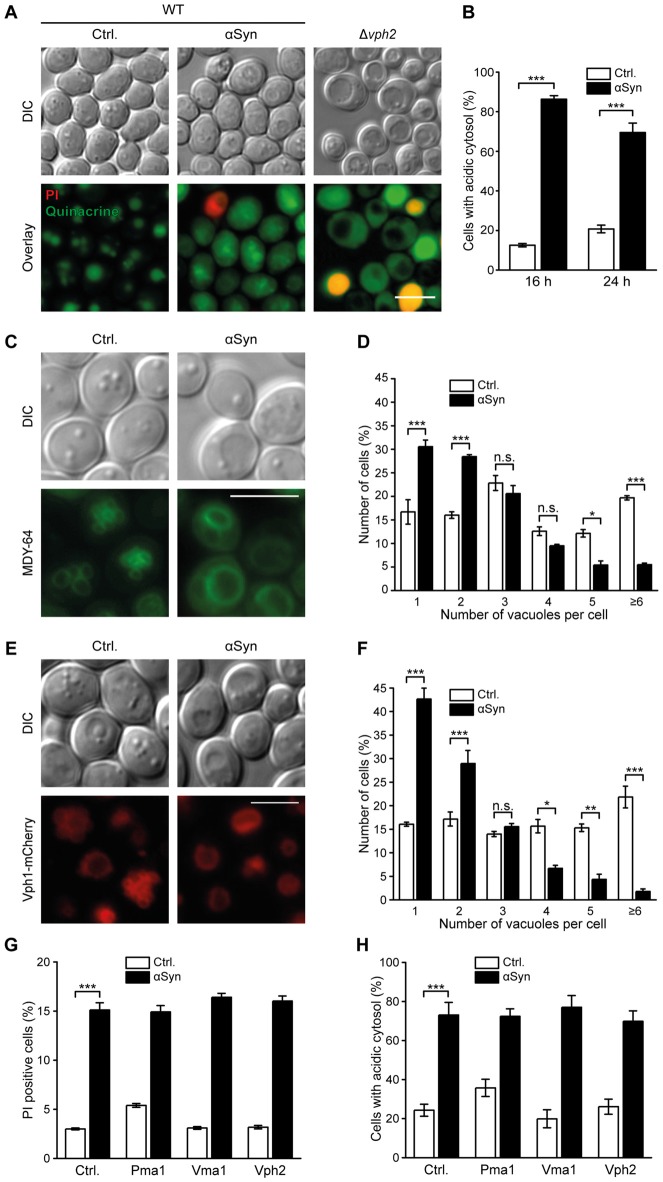
αSyn expression causes cytosolic acidification and alterations of vacuolar morphology. **(A,B)** Quinacrine staining of wild type (WT) cells expressing human αSyn for 16 h and 24 h, respectively, or harboring the empty vector control (Ctrl.), as well as of Δ*vph2* cells, to visualize acidic cell organelles. Representative micrographs after 16 h of expression **(A)** and quantification of cells with an acidic cytosol at 16 h and 24 h **(B)** are displayed. Representative micrographs after 24 h of expression are shown in Supplementary Figure S2A. Counterstaining with PI was performed to exclude dead cells from the analysis. For each strain 400–600 cells were evaluated. Means ± SEM; *n* = 3. **(C–F)** Analysis of vacuolar morphology of cells expressing αSyn for 16 h or harboring the empty vector control either via MDY-64 staining **(C,D)** or upon visualization of the vacuolar membrane via a chromosomally mCherry-tagged version of Vph1 **(E,F)**. Representative micrographs **(C,E)** and quantification of cells containing the depicted number of vacuoles **(D,F)** are shown for each approach. MDY-64 stained cells were counterstained with PI to exclude dead cells from the quantification. For each strain 350–650 cells were evaluated. Means ± SEM; *n* = 3. **(G)** Flow cytometric quantification of loss of membrane integrity, indicated by PI staining of cells expressing αSyn with or without co-expression of indicated proteins for 24 h or harboring the corresponding empty vector controls. Means ± SEM; *n* = 9. **(H)** Quantification of cells with an acidic cytosol analyzed via quinacrine staining of cells as described in **(G)**. Representative micrographs are shown in Supplementary Figure S2C. Cells were counterstained with PI to exclude dead cells. Means ± SEM; *n* = 3. n.s. not significant, *p < 0.05, **p < 0.01 and ***p < 0.001; Scale bar represents 5 μm.

As the acidic pH in the vacuolar lumen is essential for proper function of Pep4 (Sørensen et al., 1994), disturbances of pH homeostasis might contribute to the reduction in Pep4 activity observed upon αSyn expression. Therefore, we visualized acidic cellular compartments via quinacrine staining after 16 h and 24 h of αSyn expression and used PI counterstaining to exclude dead cells from the analysis. While quinacrine accumulated within the vacuoles of cells harboring the vector control, αSyn-expressing cells mostly exhibited a rather diffuse fluorescence signal throughout the cell, with some cells displaying only a weak vacuolar quinacrine staining (Figures 2A,B and Supplementary Figure S2A). A mutant completely defective in vacuolar acidification due to a lack of Vph2, the assembly factor of the vacuolar H^+^-ATPase (V-ATPase), an evolutionary conserved multimeric protein complex that shuttles protons into the vacuole (Li and Kane, 2009), completely lost any vacuolar quinacrine staining (Figure 2A). Thus, the expression of αSyn resulted in clear cytosolic acidification, while the pH of the vacuoles seemed only slightly affected, indicated by decreased vacuolar quinacrine intensity.

Next, we assessed a possible effect of αSyn on vacuolar morphology, since the fission-fusion equilibrium of the vacuole is also tightly connected to acidification processes of this organelle (Coonrod et al., 2013). Visualization of vacuolar membranes using either the fluorescence dye MDY-64 (Figures 2C,D) or the vacuolar membrane protein Vph1 fused to mCherry (Figures 2E,F) suggested an αSyn-mediated shift of the vacuolar fission-fusion equilibrium towards fusion, a process known to require an acidic pH of the vacuole (Ungermann et al., 1999; Coonrod et al., 2013). The percentage of cells harboring one single and enlarged vacuole increased upon αSyn expression, while cells displaying several small vacuoles decreased significantly. Exponentially growing WT cells typically contain 1–5 vacuoles of intermediate size. Progressing age or starvation has been shown to decrease the vacuolar surface-to-volume ratio (Li and Kane, 2009; Michaillat and Mayer, 2013), leading to one single, enlarged vacuole similar to the phenotype observed upon αSyn expression. To further elucidate whether this premature age-associated vacuolar shape upon αSyn expression might be due to an inhibition of fission or rather to enhanced fusion events, we monitored osmotically induced vacuolar fission upon treatment with 0.4 M NaCl (Michaillat and Mayer, 2013). Within minutes, numerous small vacuoles, visualized via Vph1-mCherry, were detectable (Supplementary Figure S2B). The presence of αSyn did not impair fission efficiency, arguing against an inhibition of this process as causal for observed changes in vacuolar morphology.

We further examined whether an enforcement of proton transport out of the cell or into the vacuolar lumen might counteract cytosolic acidification induced by αSyn. Therefore, we overexpressed components of the V-ATPase, which governs vacuolar acidification (Li and Kane, 2009), as well as the plasma membrane H^+^-ATPase Pma1, the major regulator of cytosolic pH (Martínez-Muñoz and Kane, 2008). While high levels of the V-ATPase-subunit Vma1 and the V-ATPase assembly factor Vph2 have been shown to be sufficient to increase vacuolar acidity (Hughes and Gottschling, 2012) and to extend replicative and chronological lifespan in yeast cells (Hughes and Gottschling, 2012; Ruckenstuhl et al., 2014), co-expression of these proteins did not prevent αSyn-mediated cytosolic acidification or cell death (Figures 2G,H and Supplementary Figure S2C). Furthermore, co-expression of Pma1 had no effect either. In sum, αSyn altered vacuolar proteolytic function and morphology, which was associated with a disruption of cytosolic pH homeostasis, but probably not with major changes in vacuolar acidification.

### Pep4 Counteracts αSyn-Induced Cytosolic Acidification

**Figure 3:**
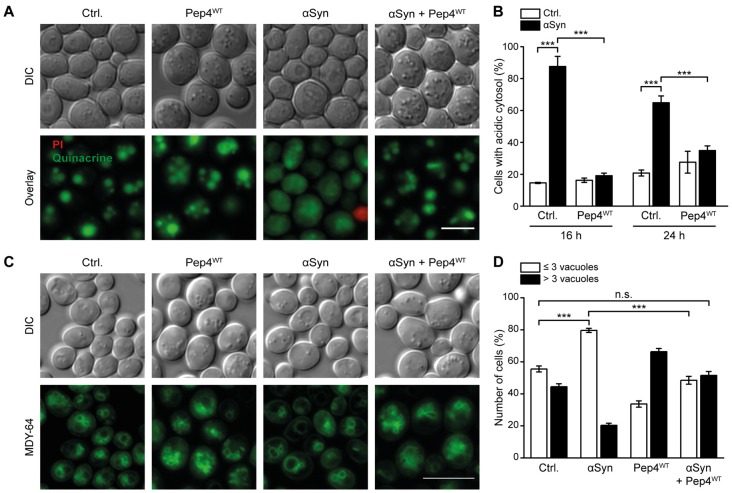
Pep4 antagonizes αSyn-induced cytosolic acidification and changes in vacuolar morphology. **(A,B)** Quinacrine staining of cells co-expressing human αSyn and Pep4^WT^ for 16 h and 24 h, respectively, or harboring the corresponding empty vector controls (Ctrl.). Representative micrographs after 16 h of expression **(A)** as well as quantification of cells with an acidic cytosol at 16 h and 24 h **(B)** are shown. Dead cells were excluded via PI staining. For each strain 500–700 cells were evaluated. Representative micrographs for 24 h after promoter induction are shown in Supplementary Figure S3A. Means ± SEM; *n* = 3. **(C,D)** MDY-64-staining to visualize vacuoles of cells as described in **(A)**. Dead cells were excluded via PI staining. Representative micrographs **(C)** and quantification of cells with less than three or with three or more vacuoles **(D)** are displayed after 16 h of expression. Data obtained at 24 h after promoter induction is displayed in Supplementary Figures S3C,D. For each strain 350–500 cells were evaluated. Means ± SEM; *n* = 3. n.s. not significant, ***p < 0.001; Scale bar represents 5 μm.

As high levels of Pep4 prevented cell death induced by αSyn, we evaluated the effects of this protease on αSyn-driven cytosolic acidification and vacuolar morphology after 16 h and 24 h of co-expression. Quinacrine staining revealed that indeed overexpression of Pep4^WT^ inhibited the αSyn-driven disturbances in pH homeostasis (Figures 3A,B and Supplementary Figure S3A). Again, this required the proteolytic activity of Pep4, as the inactive variant Pep4^DPM^ showed no effect (Supplementary Figure S3B). Moreover, the co-expression of Pep4 eliminated the effect of αSyn on vacuolar morphology observed after 16 h and restored WT organellar shape. The incidence of cells that displayed a small number (1–3) of large vacuoles, characteristic for αSyn expression, was largely reduced (Figures 3C,D). Upon entry into stationary phase, the WT vacuolar shape shifted to mostly one large vacuole per cell as expected, while Pep4-induced vacuolar fragmentation persisted (Supplementary Figure S3C). In aggregate, an increase of vacuolar hydrolytic capacity upon Pep4 overexpression was sufficient to restore cellular pH homeostasis, to correct changes in vacuolar shape and to inhibit cell death.

### Pep4 Enhances the Proteolytic Breakdown of αSyn and its Oligomers

**Figure 4:**
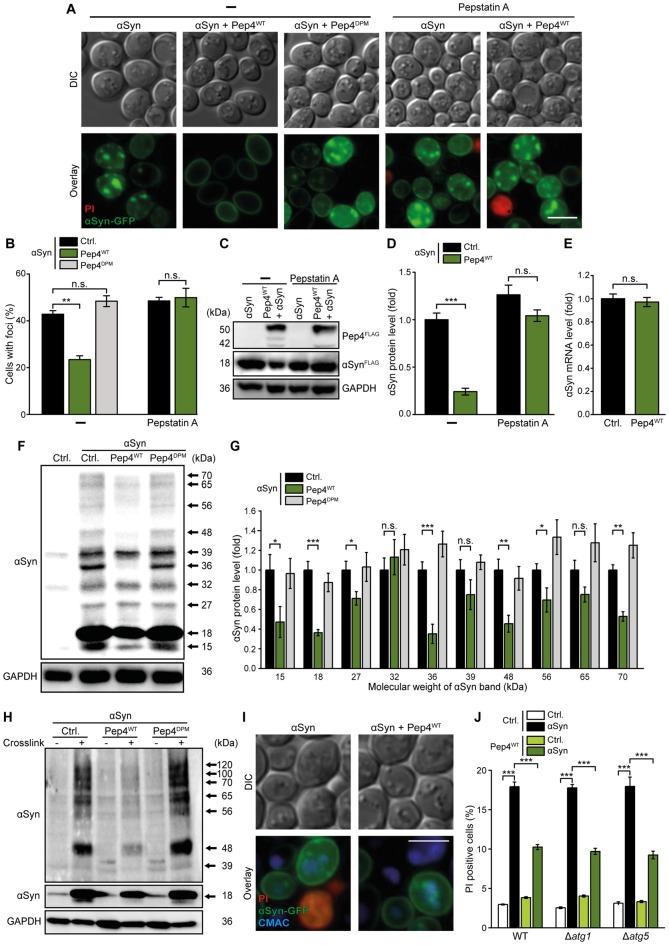
Pep4 enhances the breakdown of αSyn and its oligomers independent of autophagy. **(A,B)** Fluorescence microscopic analysis of cells expressing C-terminally GFP-tagged αSyn, co-expressing Pep4^WT^ or the inactive Pep4^DPM^ for 24 h or harboring the corresponding empty vector controls (Ctrl.). Cells were either treated with 50 μM pepstatin A or with DMSO (−). Counterstaining with PI was performed to exclude dead cells from the analysis. Representative micrographs **(A)** and quantification of cells with αSyn-foci **(B)** are shown. For each strain and treatment 200–400 cells were evaluated. For quantification of the number of foci per cell see Supplementary Figure S4A. Means ± SEM; *n* = 3. **(C,D)** Immunoblot analysis of protein extracts from cells co-expressing FLAG-tagged αSyn and Pep4^WT^ for 24 h or harboring the corresponding empty vector controls (Ctrl.). Cells were either treated with 50 μM pepstatin A or with DMSO. A representative immunoblot **(C)** and quantification of αSyn protein levels **(D)** are displayed. Blots were probed with antibodies directed against FLAG epitope to detect FLAG-tagged αSyn and Pep4^WT^ and against GAPDH as loading control. Means ± SEM; *n* = 13. **(E)** Reverse transcription quantitative PCR for determination of αSyn mRNA levels in extracts from cells as described in **(A)**. Normalization was performed using mRNA levels of *UBC6*. Means ± SEM; *n* = 8. **(F,G)** Semi-native immunoblot approach to detect αSyn oligomers in protein extracts from cells co-expressing αSyn, Pep4^WT^ or Pep4^DPM^ for 24 h or harboring the corresponding empty vector controls. A representative immunoblot **(F)** as well as densitometric quantification of detected αSyn species with indicated molecular weights **(G)** are shown. Blots were probed with antibodies directed against αSyn and against GAPDH as loading control. Means ± SEM; *n* = 8. **(H)** *In vivo* crosslinking to detect αSyn oligomers in protein extracts from cells as described above. One percent formaldehyde was used as crosslinking-reagent (+) and buffer without reagent was used as negative control (−). Blots were probed as described for semi-native immunoblots above. For a complete blot and quantification of αSyn signals please see Supplementary Figures S4B,C. **(I)** Representative micrographs of cells with C-terminally GFP-tagged αSyn, co-expressing Pep4^WT^ for 24 h. Vacuoles were stained with CMAC and PI counterstaining was performed to exclude dead cells. **(J)** Flow cytometric quantification of PI stained WT, Δ*atg1* and Δ*atg5* cells co-expressing αSyn and Pep4^WT^ for 24 h or harboring the corresponding vector controls. Means ± SEM; *n* = 8. n.s. not significant, *p < 0.05, **p < 0.001 and ***p < 0.001; Scale bar represents 5 μm.

CatD has been shown to be critically involved in the degradation of αSyn (Sevlever et al., 2008), whose oligomers are believed to be the most toxic forms of this protein (Winner et al., 2011). Given that αSyn has already been shown to form aggregates in yeast cells (Outeiro and Lindquist, 2003; Zabrocki et al., 2005), we tested whether high levels of Pep4 would reduce the abundance of αSyn-positive protein inclusions. Microscopic analysis of an αSyn-GFP fusion protein revealed αSyn aggregates in about 40% of the cells after 24 h, while only 20% of the cells co-expressing Pep4^WT^ displayed GFP-positive foci (Figures 4A,B and Supplementary Figure S4A). Again, the co-expression of the proteolytically inactive Pep4^DPM^ as well as inhibition of Pep4^WT^ via pepstatin A completely abrogated this effect. Furthermore, immunoblot analysis demonstrated that co-expression of Pep4^WT^ caused a reduction of αSyn protein levels (Figures 4C,D). This most probably reflects enforced αSyn degradation rather than decreased αSyn expression, as: (i) administration of pepstatin A prevented this effect (Figures 4C,D); and (ii) the mRNA levels of αSyn were unaltered upon co-expression of Pep4^WT^ (Figure 4E). Next, we evaluated the effect of high Pep4 levels on oligomeric αSyn species using semi-native immunoblotting. We detected the αSyn monomer (approximately 18 kDa), a smaller fragment and several oligomeric species, whose levels were significantly reduced upon co-expression of Pep4^WT^, while the proteolytically inactive variant Pep4^DPM^ had no effect (Figures 4F,G). During our experiments, samples for detection of αSyn oligomers appeared to be very sensitive to changes in temperature. Therefore, to verify the results obtained with semi-native immunoblotting, we applied *in vivo* crosslinking using 1% formaldehyde to stabilize αSyn oligomers. As expected, the cleavage product of αSyn (approximately 15 kDa), as well as oligomers with lower molecular weight were not visible with this approach, most likely due to crosslinking of these species with higher order αSyn forms (Figure 4H; a complete blot and quantifications are shown in Supplementary Figures S4B,C). However, bands with a molecular weight of about 100 and 120 kDa were detectable, which of course might not only represent pure αSyn oligomers but also stabilized interactions between αSyn and additional proteins (Figure 4H). Again, co-expression of Pep4^WT^ significantly reduced the levels of all observed αSyn species (Figure 4H and Supplementary Figures S4B,C).

There are several trafficking pathways that serve to deliver cellular material to the vacuole for subsequent proteolytic breakdown, including autophagy and the endocytic multivesicular body pathway. Microscopic analysis of αSyn-GFP cellular distribution and visualization of the vacuole with CellTracker Blue CMAC demonstrated that GFP-positive inclusions were indeed targeted to the vacuole (Figure 4I). As dysfunction of autophagy has been shown to contribute to αSyn cytotoxicity during PD (Winslow et al., 2010; Lynch-Day et al., 2012), we speculated that autophagy might be involved in the elimination of αSyn species via Pep4 and hence evaluated the requirement of a functional autophagic machinery for the observed cytoprotection. However, in strains lacking essential autophagy-related (*ATG*) genes, high levels of Pep4 still efficiently prevented αSyn-induced cell death (Figure 4J), indicating that mechanisms independent of autophagic key players are involved in the delivery of αSyn to the vacuole and subsequent Pep4-mediated degradation of αSyn species.

### Pep1 is Essential for Pep4-Mediated Cytoprotection

**Figure 5:**
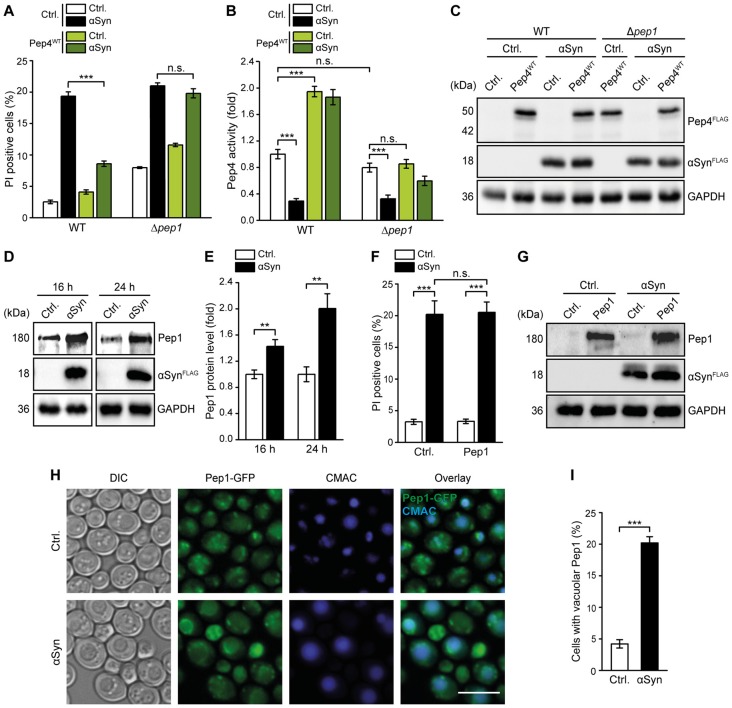
Pep1 is required for the cytoprotective effects of Pep4. **(A)** Flow cytometric quantification of loss of membrane integrity as indicated with PI staining of WT and Δ*pep1* cells co-expressing human αSyn and the Pep4^WT^ for 24 h or harboring the corresponding vector controls (Ctrl.). Means ± SEM; *n* = 4. **(B)** Measurement of Pep4 proteolytic activity in protein extracts of cells described in **(A)** 16 h after induction of expression. Values obtained for Δ*pep4* cells have been subtracted as background, followed by normalization to the average of signals from WT Ctrl. cells. Means ± SEM; *n* = 8. **(C)** Immunoblot analysis of protein extracts from cells described in **(A)**. Blots were probed with antibodies directed against FLAG epitope to detect FLAG-tagged αSyn and Pep4^WT^, and against glyceraldehyde 3-phosphat dehydrogenase (GAPDH) as loading control. **(D,E)** Immunoblot analysis of protein extracts from WT cells expressing αSyn for 16 h and 24 h or harboring the empty vector control. A representative immunoblot **(D)** and densitometric quantification **(E)** are shown. Blots were probed with antibodies against Pep1, the FLAG epitope to detect FLAG-tagged αSyn and GAPDH as a loading control. Means ± SEM; *n* ≥ 8. **(F)** Flow cytometric quantification of PI stained WT cells co-expressing αSyn and Pep1 for 24 h or harboring the corresponding vector controls. Means ± SEM; *n* = 4. **(G)** Immunoblot analysis of protein extracts from cells described in **(F)**. Blots were probed with antibodies directed against Pep1, the FLAG epitope to detect FLAG-tagged αSyn and GAPDH as loading control. **(H,I)** Fluorescence microscopic analysis of cells harboring chromosomally GFP-tagged Pep1, expressing αSyn for 24 h or harboring the corresponding vector control. Representative micrographs **(H)** and quantification of cells with Pep1 mislocalization at the vacuolar membrane **(I)** are shown. Vacuoles were counterstained with CMAC. n.s. not significant, **p < 0.001 and ***p < 0.001; Scale bar represents 5 μm.

The type I transmembrane sorting receptor Pep1 (Vps10) shuttles between the trans-Golgi network and prevacuolar endosome-like structures in order to transport vacuolar hydrolases to the vacuole (Marcusson et al., 1994; Cooper and Stevens, 1996). Interestingly, mislocalization of the mannose 6-phosphate receptor MPR300, the human ortholog of Pep1 (Whyte and Munro, 2001), has recently been shown to contribute to a reduction of CatD protein levels in SH-SY5Y cells and mice expressing αSyn (Matrone et al., 2016). To investigate the role of Pep1 in our yeast model, we co-expressed αSyn and Pep4 in a strain devoid of Pep1. PI staining revealed that Pep4-mediated cytoprotection was abolished under these conditions (Figure 5A). Furthermore, Pep4 overexpression in Δ*pep1* cells did no longer enhance Pep4 enzymatic activity, despite similar expression levels (Figures 5B,C). Interestingly, while the artificially high dosage of Pep4 obviously required Pep1 for proper activation, the endogenous Pep4 activity, as observed in cells harboring the empty vector controls, was largely unaffected by the lack of Pep1. This might be due to alternative shuttling routes in the absence of Pep1. For instance, Vth1 and Vth2, two proteins with strong similarity to Pep1, have been shown to mediate sorting of Pep4 into the vacuole to some extent (Westphal et al., 1996). Furthermore, additional pathways independent of these proteins seem to exist (Westphal et al., 1996), indicating a complex network of different routes to ensure correct localization of Pep4. Analyzing the endogenous Pep1 protein levels upon αSyn expression via immunoblotting, we observed an αSyn-induced upregulation of Pep1 levels that increased over time (Figures 5D,E). This is in line with the increased protein levels of Pep4 (Figures 1C,D) and might reflect the same compensatory mechanism in an attempt to counteract the actual lack of Pep4 activity within the vacuole. However, overexpression of Pep1 did not reduce αSyn-induced cell death (Figures 5F,G). Thus, stimulating the pathway for Pep4 trafficking into the vacuole via high levels of its sorting receptor Pep1 failed to mimic the cytoprotective effects observed upon enforced expression of Pep4. Still, Pep1 function was essential for proper activation of overexpressed Pep4 and the subsequent cytoprotection against αSyn toxicity.

As Pep1 acts as a sorting receptor for soluble vacuolar hydrolases including Pep4, efficient recycling of Pep1 from the late endosomal/prevacuolar compartments back to the trans-Golgi network is required to allow further rounds of transport. Thus, we tested for a potential effect of αSyn on the cellular distribution of Pep1 using a Pep1-GFP fusion protein expressed endogenously under its own promoter. In WT control cells, Pep1-GFP was detectable in punctate structures throughout the cell (Figure 5H), probably corresponding to endosomal compartments and the Golgi as reported previously (Seaman et al., 1997; Nothwehr et al., 1999). Upon expression of αSyn, Pep1-GFP accumulated at the limiting vacuolar membrane (Figures 5H,I). The recycling of Pep1 back to the trans-Golgi network depends on the retromer complex, and lack of components of this complex, such as Vps29 and Vps35, has been shown to cause mislocalization of Pep1 at the vacuolar membrane (Horazdovsky et al., 1997; Seaman et al., 1997; Nothwehr et al., 1999). In sum, αSyn might impair proper recycling of Pep1, which in consequence would cause insufficient sorting of Pep4 to the vacuole.

### Calcineurin is Required for the Cytoprotective Function of Pep4

**Figure 6:**
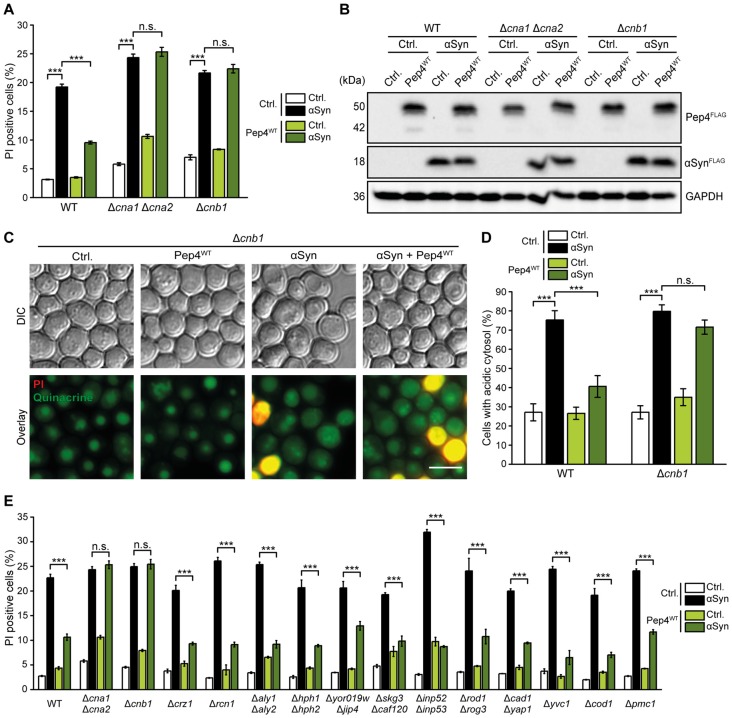
Calcineurin is essential for Pep4-mediated cytoprotection against αSyn toxicity. **(A)** Flow cytometric quantification of PI stained WT, Δ*cna1*Δ*cna2* and Δ*cnb1* cells co-expressing human αSyn and the Pep4^WT^ for 24 h or harboring the corresponding vector controls (Ctrl.). Means ± SEM; *n* ≥ 3. **(B)** Immunoblot analysis of protein extracts from cells as described above. Blots were probed with antibodies directed against FLAG epitope to detect FLAG-tagged αSyn and Pep4^WT^, and against GAPDH as loading control. **(C,D)** Quinacrine staining of WT and Δ*cnb1* cells co-expressing αSyn and Pep4^WT^ for 24 h or harboring the corresponding vector controls. Representative micrographs **(C)** and quantification of cells with an acidic cytosol **(D)** are displayed. Counterstaining with PI was performed to exclude dead cells from the analysis. For each strain 300–600 cells were evaluated. Means ± SEM; *n* = 3. **(E)** Flow cytometric quantification of PI stained WT cells and indicated mutants co-expressing αSyn and Pep4^WT^ for 24 h or harboring the corresponding vector controls. Means ± SEM; *n* = 3. n.s. not significant, ***p < 0.001; Scale bar represents 5 μm.

Cytosolic acidification has been shown to stimulate the influx of Ca^2+^ (Burns et al., 1991), and increased cytosolic Ca^2+^ levels contribute to αSyn cytotoxicity (Büttner et al., 2013a). In addition, both deletion and overexpression of the Ca^2+^/calmodulin-dependent phosphatase calcineurin increased αSyn toxicity, suggesting that moderate levels of calcineurin activity are cytoprotective (Caraveo et al., 2014). Thus, we evaluated a possible role of calcineurin in the protective effects of Pep4 and co-expressed Pep4 and αSyn in strains either devoid of both its catalytic subunits (Cna1 and Cna2) or of its regulatory subunit (Cnb1). In these cells, the toxicity of αSyn was slightly increased and, most importantly, Pep4 completely lost its protective function (Figures 6A,B). In addition, we applied quinacrine staining to analyze potential effects of dysfunctional calcineurin signaling on the disturbances of cellular pH homeostasis caused by αSyn. While αSyn-driven cytosolic acidification was largely unaffected upon lack of Cnb1, Pep4 completely lost its ability to counteract this defect (Figures 6C,D). As these data suggest an essential role for calcineurin in Pep4-mediated cytoprotection, we screened for known calcineurin targets that might contribute to the observed effects (Goldman et al., 2014). However, Pep4 co-expression efficiently protected against αSyn-mediated cell death in all deletion mutants tested, including cells lacking the main calcineurin-activated transcription factor Crz1, distinct calcineurin-regulated Ca^2+^-transporters as well as numerous candidates identified to be dephosphorylated by calcineurin via phosphoproteomics (Goldman et al., 2014; Figure 6E). For paralogous pairs of calcineurin targets, double deletion mutants were analyzed.

**Figure 7:**
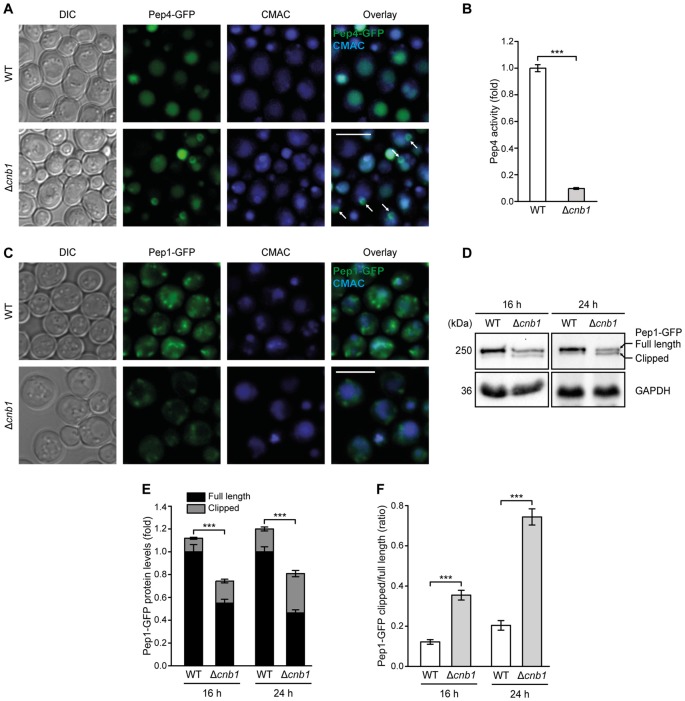
The lack of calcineurin impairs endosomal sorting of Pep1 and Pep4 and decreases Pep4 activity. **(A)** Representative micrographs of WT and Δ*cnb1* cells harboring chromosomally GFP-tagged Pep4. Vacuoles were counterstained with CMAC. White arrows indicate localization of Pep4 in prevacuolar compartments. **(B)** Measurement of Pep4 proteolytic activity in protein extracts from WT and Δ*cnb1* cells grown for 16 h. Values obtained for Δ*pep4* cells have been subtracted as background, followed by normalization to the average of signals from WT. Means ± SEM; *n* = 4. **(C)** Representative micrographs of WT and Δ*cnb1* cells harboring chromosomally GFP-tagged Pep1 grown for 24 h. Vacuoles were counterstained with CMAC. **(D–F)** Immunoblot analysis of protein extracts from cells as described in **(C)** at 16 h and 24 h, respectively. Blots were probed with antibodies directed against GFP to detect GFP-tagged Pep1 and against GAPDH as loading control. Of note, immunoblots were performed on stacked gels consisting of 7% polyacrylamide in the upper phase and 12.5% in the lower phase, respectively, to allow separation of the full length and clipped band of Pep1-GFP. Representative immunoblots **(D)** and quantification of Pep1-GFP protein levels **(E,F)** are displayed. Means ± SEM; *n* ≥ 7. ***p < 0.001; Scale bar represents 5 μm.

To test for a more direct connection between calcineurin signaling and Pep4 function, we assessed the cellular distribution of a Pep4-GFP fusion protein in cells lacking Cnb1. Pep4 accumulated in prevacuolar compartments in a small portion of Δ*cnb1* cells (Figure 7A). In line with this partially impaired delivery of Pep4 to the vacuolar lumen, the lack of functional calcineurin led to a massive decrease of proteolytic Pep4 activity (Figure 7B). Monitoring the cellular distribution of the Pep1-GFP chimera in Δ*cnb1* cells, we observed a reduction in the number of Pep1-GFP positive endosomal structures (Figure 7C). In addition, a minor accumulation of this sorting receptor at the vacuolar membrane, indicative of inefficient recycling, was detectable. Immunoblotting demonstrated that the Pep1 protein levels were decreased upon lack of Cnb1 (Figures 7D,E). Furthermore, we detected enforced proteolytic clipping at the N-terminus of Pep1 in Δ*cnb1* cells (Figures 7E,F). This phenotype has been frequently observed in cells defective in retromer-dependent recycling or the multivesicular body pathway (Marcusson et al., 1994; Cooper and Stevens, 1996; Babst et al., 2002; Balderhaar et al., 2010). Thus, the lack of functional calcineurin impairs endosomal trafficking and vacuolar targeting, however only to an extent that does not impair growth (data not shown). Under standard growth conditions, calcineurin activity is low and the resting cytosolic Ca^2+^ concentration is not affected by inactivation of calcineurin (Supplementary Figure S5B; Forster and Kane, 2000; Cyert, 2003), indicating that the impediment of endosomal trafficking and sorting in Δ*cnb1* cells is most likely not due to elevated basal Ca^2+^ levels.

**Figure 8:**
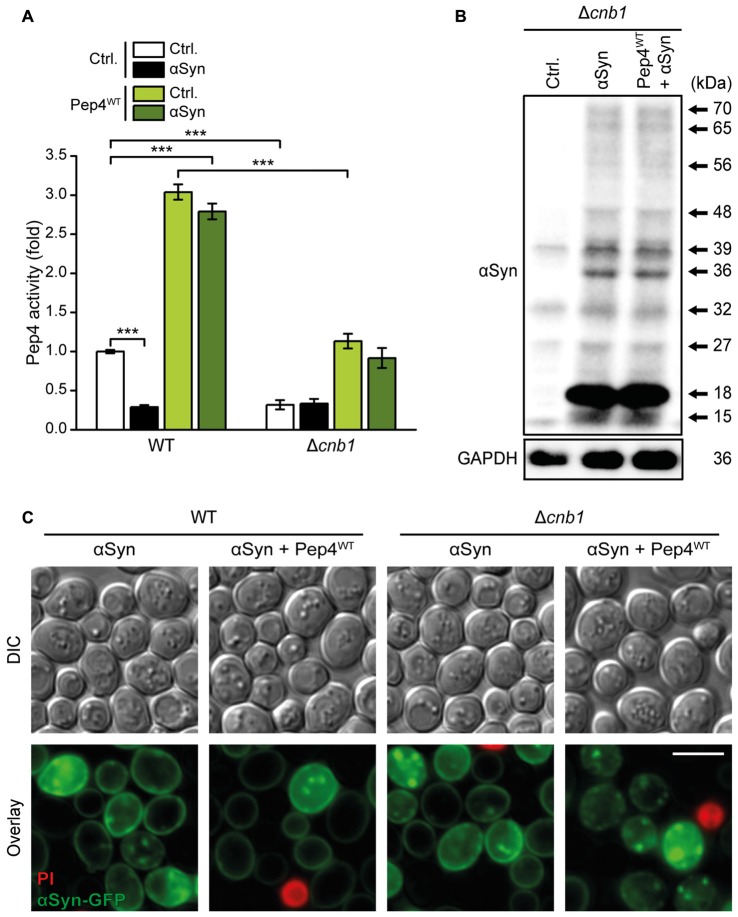
Calcineurin is essential for Pep4-mediated degradation of αSyn oligomers. **(A)** Measurement of Pep4 proteolytic activity in protein extracts from WT and Δ*cnb1* cells co-expressing αSyn and the Pep4^WT^ for 16 h or harboring the empty vector controls (Ctrl.). Values obtained for Δ*pep4* cells have been subtracted as background, followed by normalization to the average of signals from WT. Means ± SEM; *n* = 8. **(B)** Representative semi-native immunoblots to detect αSyn oligomers in protein extracts from Δ*cnb1* cells co-expressing αSyn and Pep4^WT^ for 24 h or harboring the corresponding vector controls. Corresponding densitometric quantification of detected αSyn species is shown in Supplementary Figure S5A. **(C)** Representative micrographs of WT and Δ*cnb1* cells co-expressing C-terminally GFP-tagged αSyn and Pep4^WT^ for 24 h or harboring the corresponding empty vector controls. Cells were counterstained with PI to exclude dead cells. ***p < 0.001; Scale bar represents 5 μm.

In line with hindered Pep1-mediated sorting of Pep4 to the vacuolar lumen upon lack of Cnb1, Pep4 overexpression in Δ*cnb1* cells resulted in insufficient enhancement of Pep4 activity compared to WT cells (Figure 8A). Consistently, Pep4-mediated reduction of αSyn oligomers was absent in Δ*cnb1* cells (Figure 8B, for quantification see Supplementary Figure S5A). Microscopic analysis of αSyn-GFP revealed that the lack of Cnb1 did not affect the abundance of αSyn aggregates *per se*, but prevented the decrease of these protein inclusions upon Pep4 co-expression (Figure 8C). In sum, these results indicate an essential role of calcineurin in maintaining proper Pep4 activity and in the subsequent protection against αSyn-inflicted cellular demise.

## Discussion

**Figure 9:**
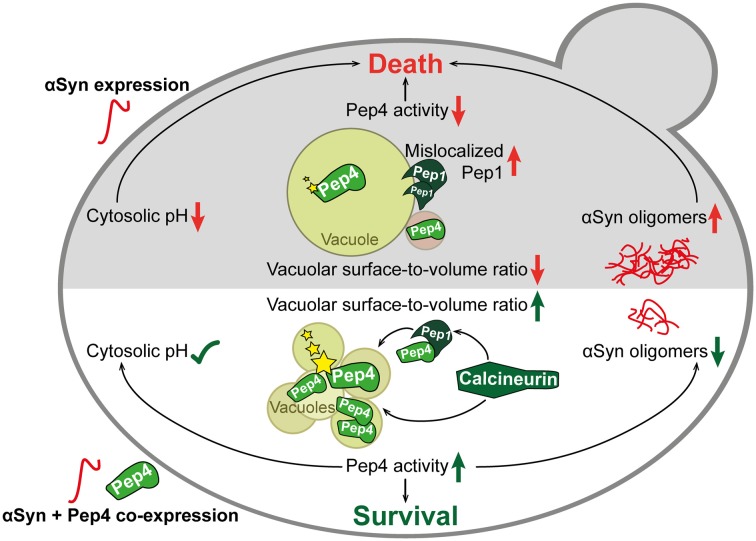
Schematic overview of αSyn toxicity and the protective interplay between calcineurin, Pep1 and Pep4. The expression of human αSyn in yeast cells (upper part in gray) led to mislocalization of Pep1 at the vacuolar membrane and of Pep4 in prevacuolar compartments. These events resulted in reduced enzymatic activity (indicated with stars) of Pep4. Furthermore, vacuolar morphology was changed upon αSyn expression, leading to a single enlarged vacuole. This was accompanied by oligomerization and aggregation of αSyn and acidification of the cytosol. Co-expression of Pep4 (lower part in white), the yeast ortholog of human cathepsin D (CatD), resulted in a stabilization of cytosolic pH, a decrease of αSyn oligomers and the formation of multiple small vacuoles, causing a higher vacuolar surface-to-volume ratio compared to cells only expressing αSyn. Ultimately, these beneficial effects of Pep4 prevented αSyn-induced cell death. Pep4-mediated cytoprotection required calcineurin, which is essential for proper localization of Pep1 and Pep4, as well as for subsequent activation of Pep4.

Malfunction of αSyn has been shown to compromise multiple cellular pathways and processes, but the molecular interplay between these mechanisms remains enigmatic. Here, we link the Ca^2+^/calmodulin-dependent phosphatase calcineurin to the enzymatic activity of the vacuolar aspartyl protease Pep4, collectively resulting in reduced αSyn toxicity in a yeast model of PD (Figure 9).

CatD presents the main lysosomal protease involved in the degradation of αSyn, and its overexpression has been shown to reduce αSyn cytotoxicity (Qiao et al., 2008; Sevlever et al., 2008; Cullen et al., 2009). Furthermore, a recent study reported a reduced enzymatic activity of CatD upon expression of αSyn (Matrone et al., 2016). In this line, we show that expression of αSyn results in a reduction of Pep4 activity in our yeast PD model and that, in turn, overexpression of Pep4 exerts cytoprotection. In addition, we observe a Pep4-mediated reduction of monomeric and oligomeric αSyn species as well as of large αSyn aggregates. Interestingly, though induction of autophagy has been shown to be sufficient for clearance of αSyn oligomers in some scenarios (Decressac et al., 2013; Hebron et al., 2013; Chen et al., 2014), the autophagic machinery does not seem to be involved in Pep4-mediated protection. Instead, prominent changes in vacuolar morphology accompanied the beneficial effects of Pep4. While cells expressing αSyn mostly exhibited one single, enlarged vacuole, a phenotype characteristic for old cells, the co-expression of Pep4 produced multiple small vacuoles, thus increasing the surface-to-volume ratio. This might contribute to the uptake of αSyn into the vacuole in an autophagy-independent manner and facilitate a more efficient proteolytic breakdown via Pep4. Furthermore, neither CatD-mediated protection of colorectal cancer cells from acetate-induced apoptosis (Oliveira et al., 2015), nor lifespan extending effects of Pep4 overexpression in yeast cells (Carmona-Gutierrez et al., 2011) are mediated via autophagy, arguing for alternative routes that contribute to the pro-survival effects of Pep4/CatD.

Recent research indicates increased CatD protein levels but reduced levels of its sorting receptor, the mannose 6-phosphat receptor MPR3000, in *post mortem* brain samples of PD patients, which might account for the observed reduction of CatD activity (Matrone et al., 2016). Reminiscent of this, we found Pep1, the yeast ortholog of MPR300, to be required for Pep4-mediated cytoprotection against αSyn toxicity. While endogenous Pep4 activity appeared unaffected in cells devoid of Pep1, probably due to alternative trafficking routes to the vacuole, the increase of proteolytic activity upon high-level expression of Pep4 observed in WT cells was absent in Δ*pep1* cells. Upon expression of αSyn, we observed a prominent mislocalization of Pep1 at the limiting vacuolar membrane. Efficient sorting of proteins from the trans-Golgi network to the vacuole requires the recycling of the sorting receptor to ensure further rounds of transport. This recycling of Pep1 has been shown to depend on the retromer, a functionally conserved multimeric protein complex (Horazdovsky et al., 1997; Nothwehr et al., 1999; Mullins and Bonifacino, 2001). Insufficient retromer function, for instance upon mutation of the core component Vps35, has been shown to impede Pep1 recycling, leading to its mislocalization at the vacuolar membrane (Seaman et al., 1997; Nothwehr et al., 1999). Notably, decreased retromer activity is an emerging scheme in the pathogenesis of PD, and mutations in Vps35 have been linked to familial PD (Zimprich et al., 2011; McMillan et al., 2017; Williams et al., 2017). Thus, it might well be that the αSyn-induced mislocalization of Pep1 is triggered by interferences with retromer-dependent Pep1 recycling, which in consequence would lead to the observed reduction in Pep4 activity.

Furthermore, hampering with the endosomal sorting and recycling system might also contribute to the prominent acidification of the cytosol upon αSyn expression. Pma1 as well as components of the V-ATPase are targeted to their location at the plasma membrane or the vacuole, respectively, via specific endosomal trafficking routes. Disturbances in endosomal protein sorting and recycling might cause insufficient targeting of those proton transporters to their destined cellular membranes. In combination, a slight decrease of proton transport out of the cell and into the vacuole would decrease the cytosolic pH. As biophysical and structural characterization of αSyn revealed an enforcement of oligomerization in an acidic environment (Uversky et al., 2001), this might play an important role in the pathophysiology of PD. Thus, αSyn would enhance its own aggregation by acidifying the cytosol.

We speculate that this might lead to a vicious cycle: αSyn impedes endosomal protein sorting, thereby causing a decrease in Pep4 activity and insufficient concentration of proton transporters at the vacuolar and plasma membranes. Thus, the cytosol starts to acidify, causing αSyn oligomers to form, which cannot be properly degraded due to insufficient vacuolar hydrolytic capacity. The resulting accumulation of αSyn amplifies the impairment of endosomal trafficking, further decreasing the available proteolytic activity in the vacuole and aggravating cytosolic acidification. This could explain why the mere overexpression of V-ATPase components or Pma1 was not capable of counteracting cytosolic acidification and cell death in the presence of αSyn: as the reduced vacuolar proteolytic capacity would still hamper proper αSyn degradation, the resulting rise in αSyn levels would further impair endosomal trafficking and thus hinder the proper targeting of the overexpressed proton transporters. In contrast, enforced expression of Pep4, even if only a small part of the artificially high levels would be properly delivered to the vacuole, could counteract the escalating levels of αSyn already early on, thereby preventing the entry into this self-amplifying loop.

In addition, Pep4 co-expression inhibited αSyn-induced cytosolic acidification, possibly via maintaining αSyn concentration below a certain threshold, which might keep the disturbances of endosomal trafficking from reaching a critical level. Furthermore, the increased vacuolar surface upon high levels of Pep4 might allow more efficient uptake of αSyn into the vacuole for subsequent proteolytic breakdown and could thus also contribute to the cytoprotective effect of Pep4. Presumably, an interplay of increased vacuolar proteolytic capacity, stabilizing effects on pH homeostasis, and alterations in vacuolar morphology mediates the cytoprotective effects of Pep4.

We identified the Ca^2+^/calmodulin-dependent phosphatase calcineurin as an essential factor in the cytoprotective mechanism of Pep4. In cells lacking Cnb1, Pep4 mislocalized to prevacuolar compartments and the endogenous Pep4 activity was largely reduced. This seems to be (at least in part) caused by malfunction of Pep1 recycling upon cargo delivery. Thus, calcineurin might contribute to processes regulating efficient endosomal recycling, allowing efficient trafficking of Pep4 to the vacuole. Of course, additional Pep1-independent processes might be involved as well, such as the maintenance of a vacuolar milieu optimal for Pep4 proteolytic function. Notably, stabilization of cellular pH homeostasis, reduction of αSyn oligomeric species and aggregates as well as inhibition of cell death achieved via high levels of Pep4 all required the presence of functional calcineurin. While our data suggests that calcineurin is required for sufficient endosomal trafficking and recycling, the direct molecular target(s) of calcineurin that mediate the observed cytoprotection remain to be identified. Neither the absence of Crz1, the main calcineurin-responsive transcription factor, nor that of distinct calcineurin-regulated Ca^2+^-channels or other downstream targets had any effect on the pro-survival effect of Pep4. The role of calcineurin in neurotoxic events is quite controversially discussed. Whereas some studies connect enhanced calcineurin activity to a reduction of cognitive function and to excitotoxicity (Foster et al., 2001; Wu et al., 2004), others describe neurotoxic consequences of prolonged pharmacological inhibition or genetic downregulation of calcineurin activity (Zeng et al., 2001; Sklar, 2006; Bahi et al., 2009). A recent study in yeast could demonstrate that both insufficient and excess calcineurin activity increased αSyn toxicity (Caraveo et al., 2014), arguing for a complex role of this signaling phosphatase in neuronal survival.

Here, we establish a so far unknown cytoprotective role for calcineurin, contributing to full enzymatic activity of yeast CatD. As both proteins are highly conserved across species barriers, this interplay might be functional in higher eukaryotes as well and thus might represent a potential new target for therapeutic strategies against PD.

## Author Contributions

AA and SB conceptualized the study; AA, LH, VK and JD performed the experiments; AA and SB analyzed the data and wrote the manuscript; DC-G, TE and WK analyzed and discussed the data and gave conceptual advice; SB supervised the study; All authors commented on the manuscript and read and approved the final version.

## Conflict of Interest Statement

The authors declare that the research was conducted in the absence of any commercial or financial relationships that could be construed as a potential conflict of interest.

## Abbreviations

CatD: cathepsin D
GAPDH: glyceraldehyde 3-phosphate dehydrogenase
HA: human influenza hemagglutinin
PD: Parkinson’s disease
PBS: phosphate buffered saline
PI: propidium iodide
SC: synthetic complete
αSyn: α-synuclein
V-ATPase: vacuolar H ^+^-ATPase
YEPD: yeast extract peptone dextrose.
