# Supplementary material for: The Coordinated Action of Calcineurin and Cathepsin D Protects Against α-Synuclein Toxicity
Source: Front Mol Neurosci. 2017 Jun 30;10:207. doi: 10.3389/fnmol.2017.00207 (PMC5491553; doi:10.3389/fnmol.2017.00207)
Supplement: Supplementary file 1 [file Data_Sheet_1.pdf]

Supplementary Material

The Coordinated Action Of Calcineurin And Cathepsin D Protects Against α-Synuclein Toxicity

Andreas Aufschnaiter[1], Lukas Habernig[1,2], Verena Kohler[1], Jutta Diessl[2], Didac Carmona-Gutierrez[1], Tobias Eisenberg[1], Walter Keller[1] and Sabrina Büttner[1,2,*]

[1] Institute of Molecular Biosciences, University of Graz, Graz, Austria
[2] Department of Molecular Biosciences, The Wenner-Gren Institute, Stockholm University, Stockholm, Sweden

* Correspondence: **Sabrina Büttner; sabrina.buettner@su.se**

Contains:

Supplementary Figures S1-S5
Supplementary Tables S1, S2

1 Supplementary Figures and Tables

1.1 Supplementary Figures

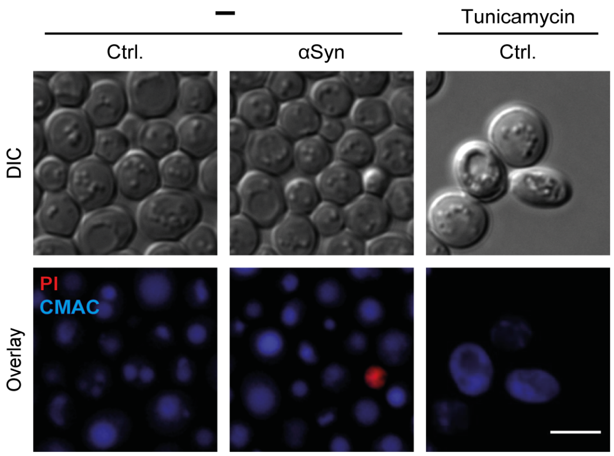

Supplementary Figure S1: αSyn expression does not result in vacuolar membrane permeabilization

Representative micrographs of cells co-expressing human α-synuclein (αSyn) and the wild type form of Pep4 ($Pep4^{WT}$) for 24 h or harboring the empty vector controls (Ctrl.). Cells were stained with CMAC to visualize vacuolar integrity and propidium iodide (PI) to exclude dead cells from the analysis. As a positive control, cells were treated with 2.5 µg/mL tunicamycin. Scale bar represents 5 µm.

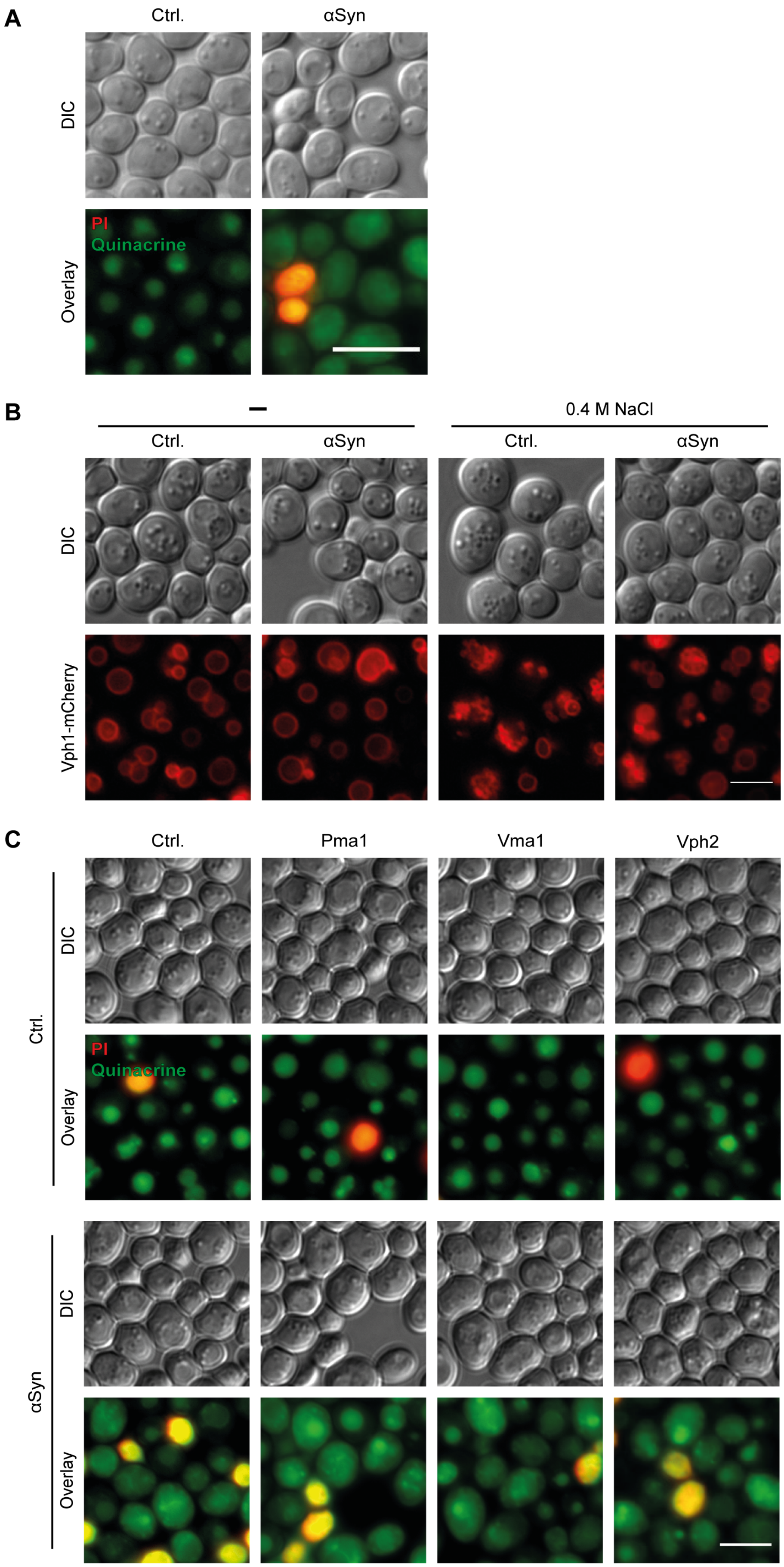
A
Ctrl.
αSyn
DIC
Overlay
PI
Quinacrine
B
–
0.4 M NaCl
Ctrl.
αSyn
Ctrl.
αSyn
DIC
Vph1-mCherry
C
Ctrl.
Pma1
Vma1
Vph2
Ctrl.
DIC
Overlay
PI
Quinacrine
αSyn
DIC
Overlay

Supplementary Figure S2: Characterization of vacuolar phenotypes upon αSyn expression

(**A**) Representative micrographs of quinacrine stained cells expressing human α-synuclein (αSyn) for 24 h or harboring the empty vector control (Ctrl.). Cells were counterstained with propidium iodide (PI) to exclude dead cells.
(**B**) Representative micrographs of cells equipped with a chromosomally mCherry-tagged version of Vph1 expressing αSyn for 24 h or harboring the empty vector control. Vacuolar morphology of untreated cells (-) and cells incubated in 0.4 M NaCl for 10 min was analyzed.
(**C**) Representative micrographs of quinacrine stained cells expressing αSyn with or without co-expression of indicated proteins for 24 h or harboring the corresponding vector control. Dead cells were excluded via PI counterstaining.
Scale bar represents 5 µm.

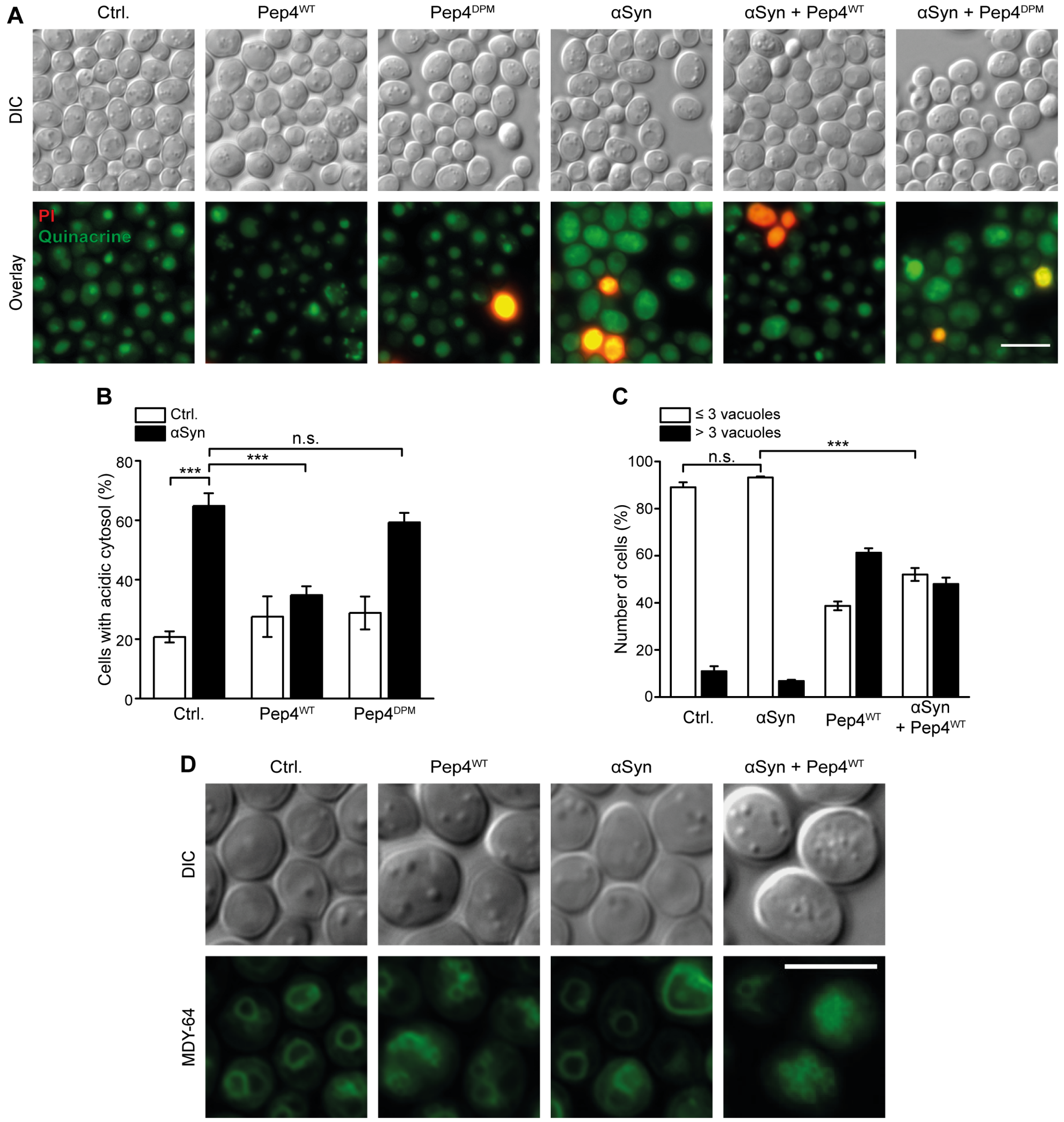

Supplementary Figure S3: Re-installation of pH homeostasis via Pep4 requires its proteolytic activity

(**A, B**) Representative micrographs (A) and quantification of cells with an acidic cytosol (B) of quinacrine stained cells co-expressing human α-synuclein (αSyn), the wild type form of Pep4 ($Pep4^{WT}$) or the enzymatically inactive form of Pep4 ($Pep4^{DPM}$) for 24 h, or harboring the empty vector controls (Ctrl.). Dead cells were excluded via propidium iodide (PI) counterstaining. Means ± SEM; n=3. For each individual experiment, 500-700 cells were evaluated.
(**C, D**) MDY-64-staining to visualize vacuoles of cells as described in (A). Dead cells were excluded via PI staining. Quantification of cells containing the indicated number of vacuoles (C) and representative micrographs (D) are displayed for 24 h after induction. Means ± SEM; n=3. For each individual experiment, 350-500 cells were evaluated.
n.s. not significant., ***$p<0.001$; Scale bar represents 5 µm.

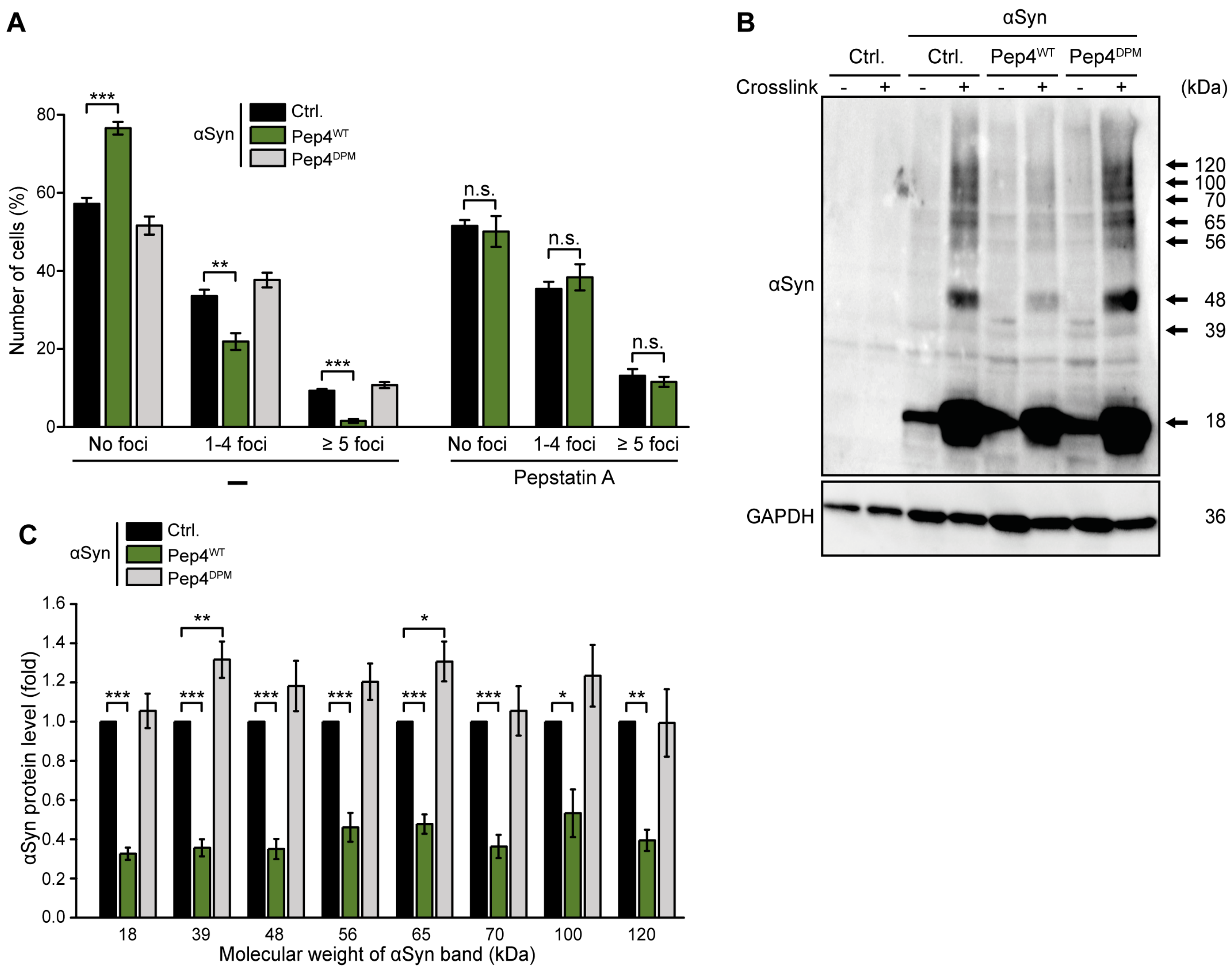

Supplementary Figure S4: Proteolytically active Pep4 increases the breakdown of αSyn and its oligomers

(**A**) Quantification of fluorescence microscopic analysis of cells expressing C-terminally GFP-tagged α-synuclein (αSyn), co-expressing wild type Pep4 ($Pep4^{WT}$) or the inactive double point mutant of Pep4 ($Pep4^{DPM}$) for 24 h or harboring the corresponding empty vector controls (Ctrl.). Cells were either treated with 50 µM pepstatin A or with DMSO (-). Counterstaining with propidium iodide (PI) was performed to exclude dead cells from the analysis. Quantification of cells containing the depicted number of foci is displayed. For each strain and treatment 200 – 400 cells were evaluated. Means ± SEM; n=3.

(**B, C**) *In vivo* crosslinking to detect αSyn and its oligomers in protein extracts from cells co-expressing αSyn and $Pep4^{WT}$, or $Pep4^{DPM}$ for 24 h or harboring the empty vector controls. One percent formaldehyde was used as crosslinking-reagent (+) and buffer without reagent was used as negative control (-). Blots were probed with antibodies directed against αSyn and glyceraldehyde 3-phosphate dehydrogenase (GAPDH) as loading control. A representative immunoblot (B) as well as quantification of αSyn bands with indicated molecular weights (C) are shown.

n.s. not significant, *$p<0.05$, **$p<0.001$ and ***$p<0.001$.

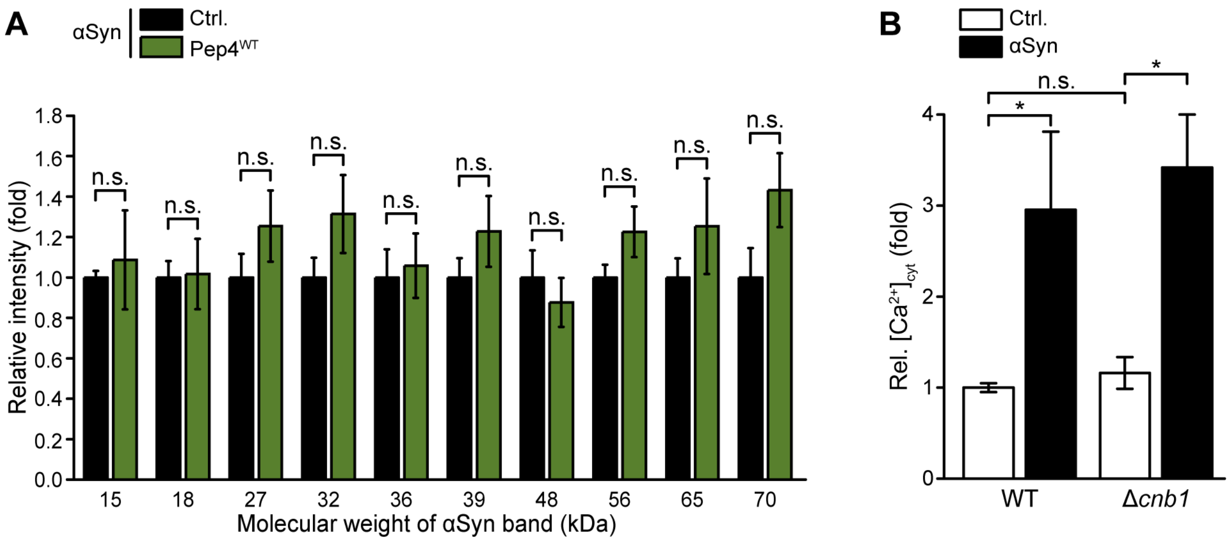

Supplementary Figure S5: The breakdown of αSyn oligomers via Pep4 requires functional calcineurin.

(**A**) Quantification of semi-native immunoblots to detect α-synuclein (αSyn) oligomers in protein extracts from Δ*cnb1* cells, co-expressing αSyn and the wild type form of Pep4 ($Pep4^{WT}$) for 24 h or harboring the corresponding vector controls (Ctrl.). Blots were probed with antibodies directed against αSyn and glyceraldehyde 3-phosphate dehydrogenase (GAPDH) as loading control. Means ± SEM; n=5.

(**B**) Aequorin-based luminescence measurement of cytosolic basal Ca^{2+} levels $[Ca^{2+}]_{cyt}$ in wild type (WT) and Δ*cnb1* cells expressing αSyn for 16 h or harboring the empty vector control. Values were normalized to OD_{600} and aequorin protein levels obtained with immunoblotting.
n.s. not significant, *$p<0.05$.

1.2 Supplementary Tables

Supplementary Table S1: Plasmids and oligonucleotides used for overexpression, gene disruption, chromosomal tagging and reverse transcription quantitative PCR.

Modification	Oligonucleotides	PCR template
Pma1 overexpression	5'- ATCTGCGGCCGCATGACTGATACATCATCCTCTTCATC -3'<br>5'- ATCTACTAGTACGGTTTCCTTTTCGTGTTGAGTAGAG- 3'	Genomic DNA
Pep1 overexpression	5'- ATCTGCGGCCGCATGATATTACTTCATTTTG -3'<br>5'- TACTGCGGCCGCCTACTGGTTTTCGTTAGATG -3'	Genomic DNA
αSyn-GFP overexpression	5'- ATATATACTAGTATGGATGTATTCATGAAAG -3'<br>5'- ATATATATCGATTTATTTGTACAATTCATCC -3'	pUG23-αSyn-GFP
CNA2 deletion	5'- TTCCCTCCCATAGAGTCACACAGGAGCCAGTACTTCTTC TTGAACCCGCAATGCGTACGCTGCAGGTCGAC -3'<br>5'- CTTACTTACTTATTGAAGTATGTACAGTGGAAATAGGAG CTTTCTCTAATCGATGAATTCGAGCTCG -3'	pFA6a–hphNT1
Control PCR *CNA2*	5'- CCGAGACAAATGAGAAAAT GTCC -3'<br>5'- GTCGACCTGCAGCGTACG-3'	
CNB1 deletion	5'- TGGTAACTCAATGGTGATCAGAATCCATAGAAGCATTTT TATTTCTTAAAATGCGTACGCTGCAGGTCGAC -3'<br>5'- CTTAAAAATATTGGCATACCATAAATGAATGAAGTGTCC CCTAGTCTTAATCGATGAATTCGAGCTCG -3'	pFA6a–hphNT1
Control PCR *CNB1*	5'- GCCGCCAAAATGGGATATA C -3'<br>5'- GTCGACCTGCAGCGTACG -3'	
CRZ1 deletion	5'- GTATTTTAGTCTCGATTGGAAGTTTCGTCAGACAGTACA AGGAAGATGCGTACGCTGCAGGTCGAC -3'<br>5'- TTATATAGAAAAAAAAAATTCCTATTCAAAGCTTAAAA AAACAAAAATAATTAATCGATGAATTCGAGCTCG -3'	pFA6a–hphNT1
Control PCR *CRZ1*	5'- GCGAGCTATATATCAGCGATAC -3'<br>5'- GTCGACCTGCAGCGTACG -3'	
ALY2 deletion	5'- CGTTGAAACATCTTTTTTTTTTTCCAGCCTTTCTCATTGT AGATCAGTCATTTTTCAAGATGCGTACGCTGCAGGTCGAC -3'<br>5'- GACGCCAGATGTAAGAACAGGAAGGGATGACGTAGGAA CCTTGTGCTAGACTAATCGATGAATTCGAGCTCG -3'	pFA6a–hphNT1
Control PCR *ALY2*	5'- CATCTCATCCCACTGTTTCATTC -3'<br>5'- GTCGACCTGCAGCGTACG -3'	
HPH2 deletion	5'- GAATAACACAAATTGATGGCAGTTTTTTACGTAGTCCAG TAGTTGTCCAGGTACAATGCGTACGCTGCAGGTCGAC -3'<br>5'- CATATGAAAAAATCACAGGATCATTTTTTGATATACAA ATACTATTTTTA ATCGATGAATTCGAGCTCG -3'	pFA6a–hphNT1
Control PCR *HPH2*	5'- GAGACCCTATTCTCATCTAC -3'<br>5'- GTCGACCTGCAGCGTACG -3'	

JIP4 deletion	5'- GGAATCTTTAAAGTACAAGGAACAGAAGATACTAAAAC ATAGGGGGAAATGCGTACGCTGCAGGTCGAC -3'<br>5'- GTTATAGAATTATATCGAATAAACACATAAGAACGTAA GACCAACTA ATCGATGAATTCGAGCTCG -3'	pFA6a–hphNT1
Control PCR *JIP4*	5'- GAGTGGCTTCTTCTAGGTCTC -3'<br>5'- GTCGACCTGCAGCGTACG -3'	
CAF120 deletion	5'- CGGTTTCCCCACACAAAGAACACGTTACTTGGCAAATTC AGCTCTTATGCGTACGCTGCAGGTCGAC -3'<br>5'- CTTTTCTTAATTGTCTCTCTTTTTGTAAAATTACAACCA GTCGGTCGAGTTAATCGATGAATTCGAGCTCG -3'	pFA6a–hphNT1
Control PCR *CAF120*	5'- GCCACAGAAAACTTACTGGAG -3'<br>5'- GTCGACCTGCAGCGTACG -3'	
INP53 deletion	5'- GAAAATAACTGGGGCGAAGAATATCTAGTTATCCACTCC TTCATAGAATGCGTACGCTGCAGGTCGAC -3'<br>5'- GGGATACAAACGGAACAACAACCACACTTCAAAGATAA CATATTCTCAATCGATGAATTCGAGCTCG -3'	pFA6a–hphNT1
Control PCR *INP53*	5'- GGGGATGTTCTACTGGCAGA -3'<br>5'- GTCGACCTGCAGCGTACG -3'	
ROG3 deletion	5'- CATAGAGGCAGCTCTCTTAGCAAAATAAAAATACAAAA AGTTCGACATGCGTACGCTGCAGGTCGAC -3'<br>5'- ATATGAAACTATACAAGCTTAATGCACGAGCCGAAACA ATATCGGCGACTAATCGATGAATTCGAGCTCG -3'	pFA6a – hphNT1
Control PCR *ROG3*	5'- CTGCCTTCCGTACGTCACAA -3'<br>5'- GTCGACCTGCAGCGTACG -3'	
YAP1 deletion	5'- GTTTTTTGCCACCCAAAACGTTTAAAGAAGGAAAAGTTG TTTCTTAAACCATGCGTACGCTGCAGGTCGAC -3'<br>5'- CATTATAGAAAAAGTTCTTTCGGTTACCCAGTTTTCCAT AAAGTTCCCGCTTTAATCGATGAATTCGAGCTCG -3'	pFA6a – hphNT1
Control PCR *YAP1*	5'- CGGAAACGGCAGTAAACGAC -3'<br>5'- GTCGACCTGCAGCGTACG -3'	
YVC1 deletion	5'- CAATAGAATTCAGTTATAAAATATAATATTACTAGAAC AGGAGCATTATGCGTACGCTGCAGGTCGAC -3'	pFA6a – hphNT1
Control PCR *YVC1*	5'- TCATGCAGCATTCCGGTCGC -3'<br>5'- GTCGACCTGCAGCGTACG -3'	
COD1 deletion	5'- GACATAGTTGACATATCAGACCTACAGAAACATAGGAA TCGGTAAATGCGTACGCTGCAGGTCGAC -3'<br>5'- GTAATATAAGTATATAAATACAAAAAGGGGTACTACAT AAAAGATTTATCAATCGATGAATTCGAGCTCG -3'	pFA6a – hphNT1
Control PCR *COD1*	5'- CAGGAATGTTTACTAAAAGAC -3'<br>5'- GTCGACCTGCAGCGTACG -3'	
PEP1 deletion	5'- CTCTGTATATCTGGAAAAGCCCTGAAGTGTCCAGTAGT CATCACACGTTATGCGTACGCTGCAGGTCGACC -3'<br>5'- GTTTATGAAAAGTATATGGAATTATCTACTCTATGTAA AGTAATCTCTCTAATCGATGAATTCGAGCTCG -3'	pFA6a – hphNT1
Control PCR *PEP1*	5'- CGCCCCTTGAATGAACACC -3'<br>5'- GTCGACCTGCAGCGTACG -3'	

C-terminal tagging of *PEP4* with HA or GFP	5'- AGCTCTCTAGATGGCAGAAAAGGATAGGGCGGAGAAGT AAGAAAAGTTTAGCTCAATCGATGAATTCGAGCTCG -3'<br>5'- ATACTATTCTATTTACGATTTGGGCAACAATGCGGTTGG TTTGGCCAAAGCAATTCGTACGCTGCAGGTCGAC -3'	pYM16 or pYM 25
Control PCR *PEP4*	5'- ACACTTCAAAGGATACTGAA -3'<br>5'- GTCGACCTGCAGCGTACG -3'	
C-terminal tagging of *PEP1* with GFP	5'- GTTTATGAAAAGTATATGGAATTATCTACTCTATGTAAA GTAATCTCTCTAATCGATGAATTCGAGCTCG -3'<br>5'- GAGGAAAACATCGACAGGCCTGATTCTACAGCGCCATCT AACGAAAACCAGCGTACGCTGCAGGTCGAC – 3'	pYM25
Control PCR *PEP1*	5'- CGAGCTCGAATTCATCGAT -3'<br>5'- ATAGCATGTGCATCGTCCAC -3'	
C-terminal tagging of *VPH1* with mCherry	5'- TGAGTATAAAGACATGGAAGTCGCTGTTGCTAGTGCAA GCTCTTCCGCTTCAAGCCGTACGCTGCAGGTCGAC –3'<br>5'- ATTATTTAATGAAGTACTTAAATGTTTCGCTTTTTTTA AAAGTCCTCAAAATTTAATCGATGAATTCGAGCTCG -3'	pCM79-pFA6a-3mCherry-hphNT1
Control PCR for *VPH1* tagging	5'- TGGGTGTGTTTATGACGGT -3'<br>5'- CAAGACTGTCAAGGAGGG -3'	
q-RT-PCR αSyn	5'- AGAAGACAGTGGAGGGAGCA -3'<br>5'-TGTCAGGATCCACAGGCATA -3'	
q-RT-PCR *PEP1*	5'– ATTGGATGCTCCCTCATCAC -3'<br>5'- TATACAAACCATGCGGCAAA - 3'	
q-RT-PCR *PEP4*	5'- TTGCTGCAAAAGTCCACAAG -3'<br>5'- TTCGGGGTTAGCTTTCTCAA -3'	
q-RT-PCR *UBC6*	5'- ACCCTGATACTTGGA -3'<br>5'- GTCTTCTTCTGATGG -3'	

Supplementary Table S2: Strains used in this study

Strain	Genotype	Source
BY4741 (WT)	*MAT***a**; *his3*Δ1; *leu2*Δ0; *met*15Δ0; *ura3*Δ0	Euroscarf
Δ*cna1*	BY4741 *cna1*Δ::kanMX4	Euroscarf
Δ*cna1*Δ*cna2*	BY4741 *cna1*Δ::kanMX4; *cna2*Δ::hphNT1	This study
Δ*cnb1*	BY4741 *cnb1*Δ::hphNT1	This study
Δ*crz1*	BY4741 *crz1*Δ::hphNT1	This study
Δ*rcn1*	BY4741 *rcn1*Δ::kanMX4	Euroscarf
Δ*aly1*	BY4741 *aly1*Δ::kanMX4	Euroscarf
Δ*aly1*Δ*aly2*	BY4741 *aly1*Δ::kanMX4; *aly2*Δ::hphNT1	This study
Δ*hph1*	BY4741 *hph1*Δ::kanMX4	Euroscarf
Δ*hph1*Δ*hph2*	BY4741 *hph1*Δ::kanMX4; *hph2*Δ::hphNT1	This study
Δ*yor019w*	BY4741 *yor019w*Δ::kanMX4	Euroscarf
Δ*yor019w*Δ*jip4*	BY4741 *yor019w*Δ::kanMX4; *jip4*Δ::hphNT1	This study
Δ*skg3*	BY4741 *skg3*Δ::kanMX4	Euroscarf
Δ*skg3*Δ*caf120*	BY4741 *skg3*Δ::kanMX4; *caf120*Δ::hphNT1	This study
Δ*inp52*	BY4741 *inp52*Δ::kanMX4	Euroscarf
Δ*inp52*Δ*inp53*	BY4741 *inp52*Δ::kanMX4; *inp53*Δ::hphNT1	This study
Δ*rod1*	BY4741 *rod1*Δ::kanMX4	Euroscarf
Δ*rod1*Δ*rog3*	BY4741 *rod1*Δ::kanMX4; *rog3*Δ::hphNT1	This study
Δ*cad1*	BY4741 *cad1*Δ::kanMX4	Euroscarf
Δ*cad1*Δ*yap*1	BY4741 *cad1*Δ::kanMX4; *yap1*Δ::hphNT1	This study
Δ*yvc1*	BY4741 *yvc1*Δ::hphNT1	This study
Δ*cod1*	BY4741 *cod1*Δ::hphNT1	This study
Δ*pmc1*	BY4741 *pmc1*Δ::hphNT1	Euroscarf
Δ*pep4*	BY4741 *pep4*Δ::kanMX4	Euroscarf
Δ*atg1*	BY4741 *atg1*Δ::kanMX4	Euroscarf
Δ*atg5*	BY4741 *atg5*Δ::kanMX4	Euroscarf
Δ*vph2*	BY4741 *vph2*Δ::kanMX4	Euroscarf
